# Synaptotagmin-7 drives stress-induced cardiomyocyte necroptosis via the p53-Bak-mPTP axis

**DOI:** 10.7150/thno.119528

**Published:** 2026-01-01

**Authors:** Jialei Li, Shuang Wang, Yu Han, Jinhong Liu, Yuhan Li, Jingyu Zhang, Liping Guo, Yue Jin, Jiasong Chang, Ting Liu, Lan Zhou, Siqi Liu, Guangzhao Yang, Shengxiao Zhang, Jimin Cao, Jie Na, Teng Sun

**Affiliations:** 1 MOE Key Laboratory of Cellular Physiology, and the Department of Physiology, School of Basic Medicine, Shanxi Medical University, 56 Xinjiannan Road, Taiyuan, Shanxi 030001, China.; 2 Department of Cardiology, Shanxi Cardiovascular Hospital, 18 Yifen Street, Taiyuan, Shanxi 030024, China.; 3 Shanxi Bethune Hospital, 99 Longchengda Street, Taiyuan, Shanxi 030032, China.; 4 First Hospital of Shanxi Medical University, 85 Jiefangnan Road, Taiyuan, Shanxi 030001, China.; 5 Second Hospital of Shanxi Medical University, 382 Wuyi Road, Taiyuan, Shanxi 030001, China.; 6 SXMU-Tsinghua Collaborative Innovation Center for Frontier Medicine, Shanxi Medical University, 56 Xinjiannan Road, Taiyuan, Shanxi 030001, China.; 7 Center for Regeneration, Aging and Chronic Diseases, School of Basic Medical Sciences, Tsinghua University, 30 Shuangqing Road, Beijing 100084, China.; 8 State Key Laboratory for Complex, Severe and Rare Diseases, Tsinghua University, 30 Shuangqing Road, Beijing 100084, China.

**Keywords:** Synaptotagmin-7, stress-induced myocardial injury, necroptosis, mPTP, p53, Bak

## Abstract

**Rationale:** High-intensity psychological and physiological stress contributed greatly to development of cardiac disorders in contemporary society. However, the underlying molecular mechanisms remain largely unknown. Synaptotagmin-7 (Syt7), a Ca²⁺ sensor with high affinity, has been associated with synaptic transmission and tumor progression, but its role in cardiac stress responses remains poorly defined.

**Methods:** Corticosterone (CORT) was used to induce stress injury *in vivo* and *in vitro.* The expression of Syt7 was modulated by genetic knockout, injection of adenoviral siRNA or injection of adeno-associated virus serotype 9 (AAV9) shRNA. Cardiac function and remodeling were evaluated by echocardiography, electrocardiography, and histological staining. Necroptosis was analyzed by propidium iodide (PI) staining, lactate dehydrogenase (LDH) release detection, and necroptosis marker levels. Ca²⁺ overload, ROS production, mitochondrial permeability transition pore (mPTP) opening, and bioenergetic profiling were assessed to evaluate mitochondrial function. Co-IP assay was performed to detect protein interaction, and ChIP- qPCR was performed to assess transcriptional regulation.

**Results:** Syt7 expression was significantly upregulated in both cardiomyocytes and heart tissues exposed to CORT. Both genetic knockout and cardiomyocyte-specific knockdown of Syt7 significantly preserved cardiac function and rhythm, and alleviated myocardial hypertrophy and fibrosis in CORT-treated mice. Mechanistically, Syt7 regulated necroptosis by promoting calcium overload, ROS production, mitochondrial ΔΨm dissipation, and mPTP prolonged opening. Notably, Syt7 interacted with transcription factor p53 and enhanced p53- mediated transcription of *Bcl-2 homologous antagonist/killer* (*Bak*). Syt7, p53 and Bak constitute a novel signaling axis to regulate mitochondrial dysfunction and necroptosis. Therapeutically, cardiac delivery of Syt7-targeting siRNA via adenoviral vectors significantly alleviated structural remodeling, electrophysiological instability, and myocardial necrosis in CORT-challenged mice.

**Conclusions:** The study identified Syt7 as a novel upstream regulator involved in cardiomyocyte necroptosis triggered by stress stimuli through a p53-Bak-mPTP pathway. Therapeutic targeting of Syt7 offers a promising strategy for protecting the heart against psychological or neuroendocrine stress-related injury.

## Introduction

Long-term or repeated exposure to natural stressors, such as earthquakes, human-made disasters (e.g., terrorist attacks and wars) 1, 2, economic stressors (e.g., work pressure and social isolation) 3, life factors (e.g., childhood trauma and marital problems) 4, and other stressors like noise 5, 6, can severely damage the cardiovascular system. A broad range of systematic reviews and population—based investigations—have established that psychological stress contributes not only to the development of cardiovascular disease but also significantly predicts mortality, across general populations, individuals at elevated cardiovascular risk, and patients with confirmed diagnoses 7-10. Stress contributes to a range of acute clinical cardiac events—such as arrhythmias (e.g., ventricular tachycardia, fibrillation), myocardial infarction, and other acute coronary syndromes—via the activation of autonomic, neuroendocrine, metabolic, and immune pathways 11-13. Studies have shown that stress exposure in zebrafish and mice significantly reduces cardiomyocyte division, disrupts myocardial growth and trabecular architecture, and ultimately triggers maladaptive hypertrophy and impaired systolic performance during early cardiac development 14. Furthermore, cold stress has been shown to induce mitochondrial damage in mice, resulting in abnormalities in cardiac structure and function 15. Nonetheless, the molecular pathways driving myocardial damage in response to stress have yet to be fully elucidated.

Several types of cardiomyocyte death have already been demonstrated in stress-induced cardiac injury, including apoptosis 16, autophagy 15, and ferroptosis 15. Necroptosis (also known as programmed necrosis), exhibits tightly regulated caspase-independent form of necrotic cell death 17, and contributes greatly to stress-induced cell injury. Necroptosis-related target genes have been shown to be significantly upregulated in the stress-induced hippocampus and brain dysfunction in mice 18, 19. In addition, necroptosis is activated in stress-induced mouse models of depression and in human astrocyte injury models induced by corticosterone (CORT) 20. One of the major regulatory pathways of necroptosis is mitochondrial permeability transition pore (mPTP)-mediated necroptosis. mPTP functions as a voltage-dependent, non-selective channel that facilitates the diffusion of small solutes (≤1.5 kDa). Persistent opening disrupts mitochondrial homeostasis by dissipating membrane potential and cristae structure, promoting organelle swelling and cessation of ATP generation 21. Sustained mPTP permeability facilitates unregulated translocation of cytosolic components into the mitochondrial interior, provoking osmotic imbalance, depolarization of the mitochondrial membrane, bioenergetic dysfunction, and progression toward necrotic cell death 22. However, necroptosis, especially mitochondrial pathway of necroptosis, has not yet been reported in stress-induced cardiac injury.

Synaptotagmin-7 (Syt7), a Synaptotagmin family member, features two C2 domains that binds Ca^2+^ with strong affinity, enabling it to regulate vesicle fusion in calcium signals 23. The functional significance of Syt7 has been widely examined in the context of calcium-mediated synaptic transmission, where it modulates various modes of vesicle release, including asynchronous discharge following action potentials, short-term plasticity-driven rapid release, and baseline synaptic fusion events 24, 25. Based on these functions, Syt7 participates in a variety of neurological disorders including manic-like behavioral abnormalities 26 and cognitive impairment 27. Syt7 is implicated to tumorigenesis, such as chronic lymphocytic leukemia 28, NSCLC 29, gastric cancer 30, thyroid carcinoma 31 and HNSCC 32. Recent advances suggest that Syt7 is abundantly expressed at cardiac sympathetic nerve terminals and contributes to blood pressure regulation 33, 34. Moreover, Syt7 has been implicated in hypoxia/reoxygenation (H/R)-induced myocardial injury 35. Our previous work demonstrated that Syt7 is abundant in the heart and promote myocardial hypertrophy by regulating autophagy 36. However, the role of Syt7 in cardiovascular biology remains limited. In particular, it is unclear whether Syt7 contributes to the development of stress-induced myocardial injury and, if so, through which specific molecular mechanisms.

Our present work identifies Syt7 as a novel regulator in stress-induced myocardial injury. Syt7 is upregulated in response to CORT. Loss-of-function of Syt7 preserves cardiac function and attenuates stress-induced arrhythmias, myocardial hypertrophy, and fibrosis. Mechanistically, Syt7 promotes necroptosis and mitochondrial dysfunction by interacting with p53 and enhancing its transcription of *Bak*. Importantly, adenoviral-mediated knockdown of *Syt7* (si-*Syt7*) confers significant cardioprotective effects, providing mechanistic support for the development of Syt7-directed therapies to mitigate stress-driven myocardial injury.

## Materials and Methods

### Establishment of *Syt7* knockout mouse and stress-induced myocardial injury model

*Synaptotagmin-7* (*Syt7*) knockout (KO) mice were purchased from Cyagen Biosciences. DNA was extracted from tail clippings at four weeks of age, and the mice were genetically identified by PCR employed the primers: Forward primer: 5'-GAGTTGAGTTAATGTGGGCAG-3'; Reverse primer 1: 5'-CAAGGTGCTTAGTCTATGGAC-3'; Reverse primer 2: 5'-ATAGCCAGTGATTGCAGTATGG-3'. To control for hormonal variability, male *Syt7* KO and littermate wild-type (WT) mice (8 weeks, 24-26 g) were administered corticosterone (CORT) (10 mg/kg/day, i.p.) for seven days to induce stress injury 37. Control groups received saline in the same volume.

### Construction and infection of adenovirus

*Syt7* siRNA consisted of 5'-GCAAACTGGGCAAACGCTACA-3' (sense), and 5'-TGTAGCGTTTGCCCAGTTTGC-3'(antisense). The scrambled siRNA was 5'-ACTACCGTTGTTATAGGTG-3' (sense), and 5'-CACCTATAACAACGGTAGT-3' (antisense). The ADV3 adenoviral vector (GenePharma, China) was used to be cloned with siRNAs or the corresponding scrambled controls. The adenovirus carrying *Syt7* siRNA (Ad-si*Syt7*) or scramble control (Ad-siNC) was diluted to 9 × 10^9^ PFU/mL. A total of 45 μL of adenoviral solution was administered into the mouse myocardium at three distinct injection sites for *in vivo* gene delivery. For *in vitro* knockdown, cells received adenoviral transduction with Ad-si*Syt7* or Ad-siNC at an MOI of 80 after reaching 70-80% confluency. Cells were incubated for 48 h before subsequent analyses.

### AAV9 virus preparation and *in vivo* administration

To achieve cardiomyocyte-specific knockdown of *Syt7* in C57BL/6J mice, short hairpin RNA (shRNA) sequences targeting *Syt7* (sense: 5′-GCAAACTGGGCAAACGCTACA-3′; antisense: 5′-TGTAGCGTTTGCCCAGTTTGC-3′) or a non-targeting scramble control were cloned into an adeno-associated virus serotype 9 (AAV9) expression vector, where their expression was driven by the cardiac-specific cTnT promoter. Recombinant AAV9 vectors (AAV9-cTnT-sh*Syt7* and AAV9-cTnT-shNC) were packaged and purified by Hanbio Biotechnology (Shanghai, China). Eight-week-old mice received *in situ* intramyocardial injections of AAV9 at a dose of 1 × 10¹¹ viral genomes (vg) per mouse.

### Echocardiography and electrocardiogram

Following the last intraperitoneal injection of CORT or saline, echocardiography and electrocardiogram were performed according to previously described methods 38. Under 2% isoflurane anaesthesia, M-mode echocardiograms of the heart were acquired using a Prospect High-Resolution Imaging System (S-Sharp, Taiwan, China) equipped with a 40 MHz probe. Prior to imaging, the mouse chest was shaved using depilatory cream and coated with a medical ultrasound coupling agent. Key echocardiographic parameters assessed included LVID;s, LVID;d, LVEDV, and LVESV. Ejection fraction (EF) and fractional shortening (FS) were derived using standard formulas to evaluate systolic function: EF = [(LVEDV - LVESV)/LVEDV] × 100%, and FS = [(LVID;d - LVID;s)/LVID;d] × 100%. Electrocardiograms (ECGs) were recorded by connecting clip wires to subcutaneous needle electrodes placed on each of the mouse limbs according to the limb lead identification on the amplifier. Baseline ECGs were recorded using the SP2006 ECG recording and analysis system (Softron, China).

### Histological staining

After echocardiography and electrocardiography, 5% isoflurane was used to induce deep anesthesia, after which cervical dislocation was performed, to ensure humane euthanasia. Mouse hearts were flushed with saline, weighed, and photographed. Additionally, the tibiae were harvested, and their length was measured. After dehydration in ascending concentrations of ethanol, the cardiac tissue was paraffin-embedded and sliced into 4 μm sections, which were then subjected to H&E staining, Masson's trichrome, and Sirius red kits following the manufacturers' protocols. These staining procedures were employed to evaluate histopathological changes, particularly myocardial vacuolation and interstitial fibrosis. TRITC-labeled wheat germ agglutinin (WGA) (Thermo Fisher, USA) was employed to label cardiomyocyte boundaries for cross-sectional area analysis.

### Cell cultivation and treatment strategy

H9c2 cells and HEK293 cells were maintained in DMEM (Hyclone, USA) containing 10% FBS, 100 U/mL penicillin, 0.1 mg/mL streptomycin, and 110 mg/mL sodium pyruvate. Neonatal rat primary cardiomyocytes were harvested from 1-to-2-day-old Sprague Dawley neonatal rats and maintained in DMEM/F-12 (Hyclone, USA) containing 5% FBS, 100 U/ml penicillin, 0.1 mg/mL streptomycin, and 0.1 mM bromodeoxyuridine. Standard culture conditions were applied (37 °C, 5% CO₂, humidified incubator). Unless otherwise indicated, cells were exposed to CORT at a dose of 1 μM for 48 h.

### PI-based visualization of necrosis

Necroptosis in myocardial tissue and cardiomyocytes was evaluated using propidium iodide (PI) (Yensea, China). PI was infused centripetally through the pulmonary artery. After 30 min, the hearts were rinsed with saline, removed, fixed in paraformaldehyde and 30% sucrose, rapidly frozen in liquid nitrogen, and sectioned at a thickness of 4 μm. Cardiomyocytes and nuclei were visualized using α-actinin and DAPI staining, respectively. H9c2 cells were exposed to PI (1.5 μM, 30 min) on ice. After fixation with 4% paraformaldehyde and DAPI staining, cells were examined by fluorescence microscopy (Nikon, Japan) to detect necrotic cardiomyocytes, and image data were recorded for analysis.

### Lactate dehydrogenase (LDH) release measurement

LDH activity was assessed in both mouse serum and cardiomyocyte culture supernatants using a kit (Nanjing Jiancheng Bioengineering Institute, China). In brief, samples were exposed to matrix buffer and coenzyme I at 37 °C for 15 min, and subsequently treated with 2,4-dinitrophenylhydrazine for another 15 min. After incubation with 0.4 M NaOH at room temperature for 5 min, optical absorbance at 450 nm was captured by a microplate spectrophotometer.

### CCK-8 assay

Cell Counting Kit-8 (CCK-8; Abbkine, BMU106-CN, China) was used to determine cells viability following the manufacturer's protocol. In brief, samples were exposed to CCK-8 solution at 37 °C for 1-3 h. The optical density at 450 nm was recorded.

### Detection of mitochondrial △Ψm

Mitochondrial Δψm was quantified using the 5,5',6,6'-tetrachloro-1,1',3,3'-tetraethyl-1H-imidazole-2-carboxylic acid (JC-1) (Beyotime, Shanghai, China) kit. Following treated with JC-1 working buffer (37 °C, 20 min), heart tissue cryosections or H9c2 cells were imaged using a fluorescence microscope (Nikon, Japan).

### Reactive oxygen species (ROS) staining

The intracellular accumulation of ROS was evaluated using a ROS detection kit (Solarbio, China). In brief, samples were incubated with 10 μM DCFH-DA at 37 ℃ for 20 min, followed by washed with serum-free medium for 3 times to remove the unincorporated dye. Afterwards, fluorescence signals were visualized using a Nikon fluorescence microscope, and signal intensity was quantified.

### Calcein-AM staining

The mitochondrial permeability transition pore (mPTP) was evaluated using the mPTP assay kit (Beyotime, China). In brief, the fluorescence quenching solution CoCl_2_ was added to the prepared Calcein AM assay buffer and mixed thoroughly. After washed with PBS, the cells were treated with the prepared solution for 30 min at 37 °C. Afterward, the medium was substituted with fresh DMEM, and the cells were maintained at 37 °C in the absence of light for another 30 min. Fluorescence signals were detected under a fluorescence microscope.

### Assessment of mitochondrial swelling

The mitochondrial swelling detection was performed as we previously described 39. Mitochondria were extracted using the mitochondrial isolation kit (Solarbio, China), and then resuspended in swelling buffer supplemented with 100 mM KCl, 5 mM KH_2_PO_4_, 50 mM MOPS, 1 mM MgCl_2_, 0.5 µM EGTA, 5 mM malate, and 5 mM glutamate. The absorbance at 520 nm (OD1) was first detected. To induce swelling, mitochondria were supplemented with 200 μM CaCl_2_, and assessed the absorbance at 540 nm (OD2) at 1 min interval for 30 min using a microplate spectrophotometer. The difference between OD2 and OD1 was used for statistical analysis.

### Dual-luciferase reporter assay

Potential transcription factor binding sites of* p53* within the promoter regions of *Bak* were predicted using the JASPAR database (http://jaspar.genereg.net/). Based on the analysis results, the *p53*-bound *Bak* promoter sequences were cloned into the pGL4 vector, downstream of the firefly luciferase gene. Cardiomyocytes were transfected with the recombinant vector using Lipofectamine 3000 (Thermo Fisher, USA). Afterwards, cells were collected and subjected to luciferase activity analysis using the Dual-Luciferase Reporter Assay Kit (HANBI, China). Firefly and Renilla luciferase signals were measured, and their activity ratio was calculated to assess reporter gene expression.

### Ca^2+^ flux assays

Intracellular Ca^2+^ levels in cardiomyocytes were assessed by the Fluo-4 NW Calcium Assay Kit (Thermo Fisher, USA). Cells were treated with Fluo-4 dye at 37 °C for 45 min, followed by an additional 30-min equilibration period at ambient temperature. Calcium flux assessment was carried out by MetaFluor software and an ion imaging system, recording the relative fluorescence intensity of calcium ions in specific cells at a frequency of once every 3 s. After the recorded curve stabilizes, 50 mM KCl was administered to excite the cells, and recording continued until the fluorescence value returned to a stable state. The relative fluorescence intensity was used to represent the relative value of intracellular calcium ions.

### Immunofluorescent staining

Cells were treated with 4% formaldehyde solution preheated to room temperature for 30 min. Subsequently, cells were rinsed 3 times with PBS, followed by permeabilization and blocking at ambient temperature for 30 min. After overnight incubation with primary antibodies anti-Syt7 (Synaptic Systems, Germany, 105173), anti-p53 (CST, USA, 32532S) at 4 °C, the cells were exposed to Alexa Fluor-labeled secondary antibodies for 1 h. Afterward, nuclei were stained with DAPI to visualize nuclear morphology. Secondary detection was performed using Alexa Fluor-conjugated goat anti-mouse IgG (Beyotime, A4028) and anti-rabbit IgG (Beyotime, A0562). Fluorescence images were acquired with a Nikon fluorescence microscope.

### Co-immunoprecipitation (Co-IP) assays

Co-IP was conducted with the Pierce® Classic IP kit (Thermo Fisher, USA). Briefly, the designated antibody was combined with protein A/G agarose slurry and gently rotated for 1 h to facilitate binding. Following cell disruption using IP lysis/wash buffer, total protein content was determined for each lysate. Next, 200 μg of protein lysate was exposed to the antibody cross-linking resin at 4 °C under continuous rotation overnight. The resulting immune complexes were isolated using magnetic separation and rinsed twice with lysis/wash buffer to eliminate non-specific proteins. Target antigens were eluted and denatured for Western blot analysis using the primary antibodies: anti-Syt7 (Synaptic Systems, Germany, 105173) and anti-p53 (CST, USA, 32532S).

### Cell bioenergetic profiling

Mitochondrial function was assessed in real time by measuring oxygen consumption rate (OCR) using the Seahorse XFe24 Analyzer (Agilent Technologies, USA), providing insight into cellular respiratory capacity. In brief, cells were exposed to Seahorse XF assay medium supplemented with 10 mM glucose, 2 mM glutamine, and 1 mM pyruvate at 37 °C for 1 h. Then, the cells were incubated with oligomycin, FCCP, and Rot/AA to assess mitochondrial respiratory parameters, including baseline oxygen consumption, ATP-linked respiration, and maximal respiratory capacity.

### Chromatin immunoprecipitation quantitative PCR (ChIP-qPCR)

ChIP Kit (Abcam, USA) was used to perform ChIP assays. Briefly, cells were fixed with formalin and glycine, followed by lysis using an ultrasonic disruptor. DNA fragments (200-1000 bp) were incubated with p53 or IgG antibody and magnetic beads. Then the DNA was eluted from the immunoprecipitated samples and analyzed by qPCR assay. Specific primers based on predicted binding sites were used: forward, 5'-CCTCTGCCTCCTGAGTGTTG-3'; reverse, 5'-TCCTGAACAGTAAAGGCCACAAG-3'. Relative DNA levels were determined by normalizing to IgG ChIP samples as negative controls.

### Western blotting

Tissues or cells were lysed with RIPA lysis buffer supplemented with a mixture of 0.1 mM PMSF and a protease inhibitor cocktail. After determining protein concentrations, lysates were denatured at 100 °C for 10 min, and subjected to SDS-PAGE. Following electrophoresis, proteins were transferred to PVDF membranes (Millipore, USA), which were then blocked using 5% skim milk for 1 h. Subsequently, membranes were incubated overnight at 4 °C with designated primary antibodies, and then exposed to HRP-labeled secondary antibodies for 1 h. Signal detection was performed using enhanced chemiluminescence (ECL) on a Bio-Rad ChemiDoc imaging system. The following antibodies were used: anti-Syt7 (Abcam, USA, ab174633; Immunoway, China, YN1931; Synaptic Systems, Germany, 105173), anti-p53 (CST, USA, #32532S), anti-Histone (Beyotime, China, AF0009), anti-Bak (CST, USA, #12105), anti-Cleaved Caspase 3 (CST, USA, #9664), anti-Cleaved Caspase 9 (CST, USA, #52873), anti-Cleaved Caspase 7 (CST, USA, #8438), anti-Bax (CST, USA, #2772), anti-Bim (CST, USA, #2933), anti-Bik (CST, USA, #4592), anti-PHOSPHO-MLKL (SER358) (ThermoFisher Scientific, USA, PA5-105678), and total and cleaved N-terminal GSDMD antibody (Abmart, China, P30823S).

### Quantitative reverse transcription-PCR

Total RNA was extracted using TRIzol reagent and reverse-transcribed into cDNA with the All-in-One First-Strand cDNA Synthesis SuperMix (AU341-02, TransGen Biotech, China). Quantitative PCR was performed using SYBR qPCR Super Mix (MF797-01, Mei5bio, China) on a QuantStudio 3 system. The mRNA levels of *PTGS2*, *Syt1*, *Syt3*, *Syt4* and *Syt7* were measured and normalized to *GAPDH*. The primers are presented as follows. Rat *PTGS2*: forward, 5'-ATGTTCGCATTCTTTGCCCAG-3'; reverse, 5'-TACACCTCTCCACCGATGAC-3'. Mouse *Syt1*: forward, 5'-CTGTCACCACTGTTGCGAC-3'; reverse, 5'-GGCAATGGGATTTTATGCAGTTC-3'. Mouse *Syt3*: forward, 5'-CTCATCTCCTCGAAGCCATAATGT-3'; reverse, 5'-CTTGGACAACAGCAAAGAGACAGA-3'. Mouse *Syt4*: forward, 5'-TGACCCGTACATCAAAATGACAA-3'; reverse, 5'-GTGGGGATAAGGGATTCCATAGA-3'. Mouse *Syt7*: forward, 5'-AACCCCTCTGCCAACTCCAT-3'; reverse, 5'-GCGGCTGAGCTTGTCTTTGT-3'. Rat* GAPDH*: forward, 5'-TGGAGTCTACTGGCGTCTT-3'; reverse, 5'-TGTCATATTTCTCGTGGTTCA-3'. Mouse *GAPDH*: forward, 5'-TGTGTCCGTCGTGGATCTGA-3'; reverse, 5'-CCTGCTTCACCACCTTCTTGA-3'.

### Statistical analysis

Data are expressed as the mean ± standard deviation (SD) based on at least three independent experiments. For comparisons between two groups, Student's *t*-test was applied. Multiple group comparisons were performed using one-way or two-way analysis of variance (ANOVA) as appropriate. Statistical analysis was conducted using GraphPad Prism software. Differences were considered statistically significant at * *p* < 0.05, ** *p* < 0.01, *** *p* < 0.001, **** *p* < 0.0001.

## Results

### Synaptotagmin-7 deficiency preserves cardiac function and alleviates stress-induced structural remodeling

**Figure 1:**
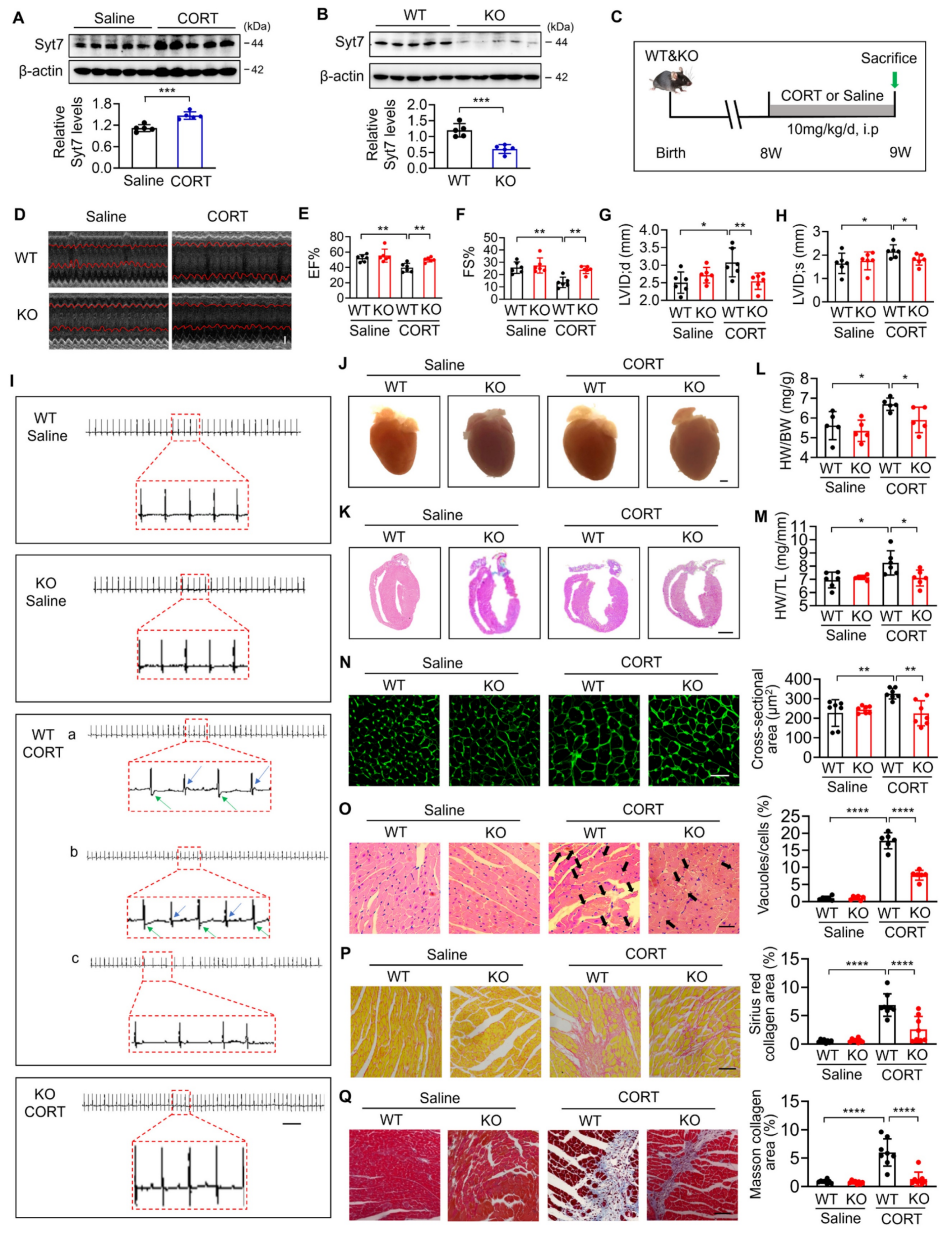
** Deletion of *Syt7* alleviates stress-induced myocardial injury in mice. A.** Western blot analysis of Synaptotagmin-7 (Syt7) protein levels in mouse cardiac tissue after intraperitoneal injection of saline or corticosterone (CORT). *** *p* < 0.001. n = 5. **B.** Representative immunoblot showing cardiac Syt7 protein expression in *Syt7* knockout (KO) mice and wild-type (WT) littermates. *** *p* < 0.001. n = 5.** C.** Schematic diagram of the experimental design. WT or *Syt7* KO mice were treated with 10 mg/kg CORT or saline daily for 7 days. **D.** Representative echocardiograms. Bar = 1 mm. **E-H.** Quantification of echocardiographic parameters: ejection fraction (EF), fractional shortening (FS), left ventricular internal diameter at diastole (LVID;d), and systole (LVID;s). * *p* < 0.05. ** *p* < 0.01. n = 6. **I.** Representative electrocardiograms. Bar = 500 ms. **J.** Representative anatomical images of mouse hearts. Bar = 1 mm. **K.** H&E staining of longitudinal cardiac sections. Bar = 2 mm. **L.** Heart weight to body weight ratio (HW/BW, mg/g). * *p* < 0.05. n = 5. **M.** Heart weight to tibia length ratio (HW/TL, mg/mm). * *p* < 0.05. n = 6. **N.** Cross-sectional area of cardiomyocytes assessed by wheat germ agglutinin (WGA) staining. Bar = 30 µm. ** *p* < 0.01. n = 7. **O.** Degree of vacuolar degeneration analyzed by H&E staining. Arrows indicate vacuolar degeneration. Bar = 40 µm. **** *p* < 0.0001. n = 6. **P-Q.** Cardiac fibrosis assessed by Sirius Red staining (P) and Masson's trichrome staining (Q), respectively. Bar = 50 µm. **** *p* < 0.0001. n = 8. Data are expressed as the mean ± standard deviation (SD).

Synaptotagmins (Syts) are key calcium sensors involved in membrane fusion and stress-responsive signaling 40-45. To explore the potential involvement of specific Syt isoforms in stress-induced cardiac injury, we analyzed the mRNA levels of *Syt1*, *Syt3*, *Syt4*, and *Syt7* in hearts of mice administered corticosterone (CORT), a primary glucocorticoid hormone widely used to model psychological stress in rodents 37. Results revealed that *Syt1*, *Syt4*, and *Syt7* were significantly upregulated upon CORT exposure, whereas *Syt3* remained unchanged (Figure S1). Among the upregulated molecules, *Syt7* displayed the most stable induction across replicates, as evidenced by the lowest standard error. A robust elevation of Syt7 protein levels was observed in CORT-treated cardiac tissue (Figure 1A). Given this expression pattern, along with the established role of Syt7 in calcium signaling and cardiac physiology, we selected it as a candidate target for in-depth functional analysis in stress-induced myocardial injury. *Syt7* knockout (KO) mice were generated through CRISPR/Cas9 (Figure 1B). At eight weeks of age, both *Syt7* KO mice and wild-type (WT) mice received intraperitoneal injections of CORT for stress induction, and cardiac function was evaluated one week later (Figure 1C). Compared to WT mice, *Syt7* KO mice exhibited improved heart function, as evidenced by increased ejection fraction (EF) and fraction shortening (FS), and restoration of left ventricular internal diameter at diastole (LVID;d) and left ventricular internal diameter at systole (LVID;s) (Figure 1D-H). Furthermore, electrocardiograms revealed that WT mice exposed to CORT developed T-wave alternans and arrhythmias, which was ameliorated by knockout of *Syt7* (Figure 1I). These findings suggest that *Syt7* deficiency mitigates the stress-induced cardiac dysfunction and arrhythmias. We further explored whether Syt7 regulates stress-induced cardiac remodeling. *Syt7* knockout mice exhibited smaller hearts following stress induction compared to control group (Figure 1J-K). Additionally, *Syt7* deficiency resulted in a significantly lower ratio of heart weight-to-body weight (HW/BW) (Figure 1L) and heart weight-to-tibia length (HW/TL) (Figure 1M). A reduction in cardiomyocyte size was also observed in the stressed hearts of *Syt7* KO mice, as assessed by wheat germ agglutinin (WGA) staining (Figure 1N). Stress induced cytoplasmic vacuolization in the myocardium, which was significantly attenuated by *Syt7* deletion (Figure 1O). Furthermore, knockout of *Syt7* mitigated cardiac interstitial fibrosis induced by CORT, demonstrated by Sirius red staining (Figure 1P) as well as Masson's trichrome staining (Figure 1Q). To achieve cardiomyocyte-specific silencing of *Syt7*, a recombinant adeno-associated virus serotype 9 (AAV9) vector encoding *Syt7*-targeting shRNA under the control of the cardiac-specific cTnT promoter was constructed and delivered via *in situ* myocardial injection (Figure S2A). Effective knockdown of *Syt7* was detected in hearts, whereas its expression remained unchanged in non-cardiac tissues, including the liver, spleen, lung, kidney, and brain (Figure S2B-G), confirming tissue specificity. Functionally, cardiac-specific silencing of *Syt7* markedly ameliorated CORT-induced cardiac dysfunction (Figure S3A-E), T-wave alternans and arrhythmias (Figure S3F). Histological analysis further demonstrated that *Syt7* knockdown mitigated CORT-induced myocardial injury including increased heart volume (Figure S4A-D), increased cardiomyocyte size (Figure S4E), cytoplasmic vacuolization (Figure S4F), and cardiac interstitial fibrosis (Figure S4G-H). Taken together, Syt7 has been identified as a critical modulator of stress-induced cardiac dysfunction and myocardial injury.

### Synaptotagmin-7 regulates cardiomyocyte necroptosis under stress

**Figure 2:**
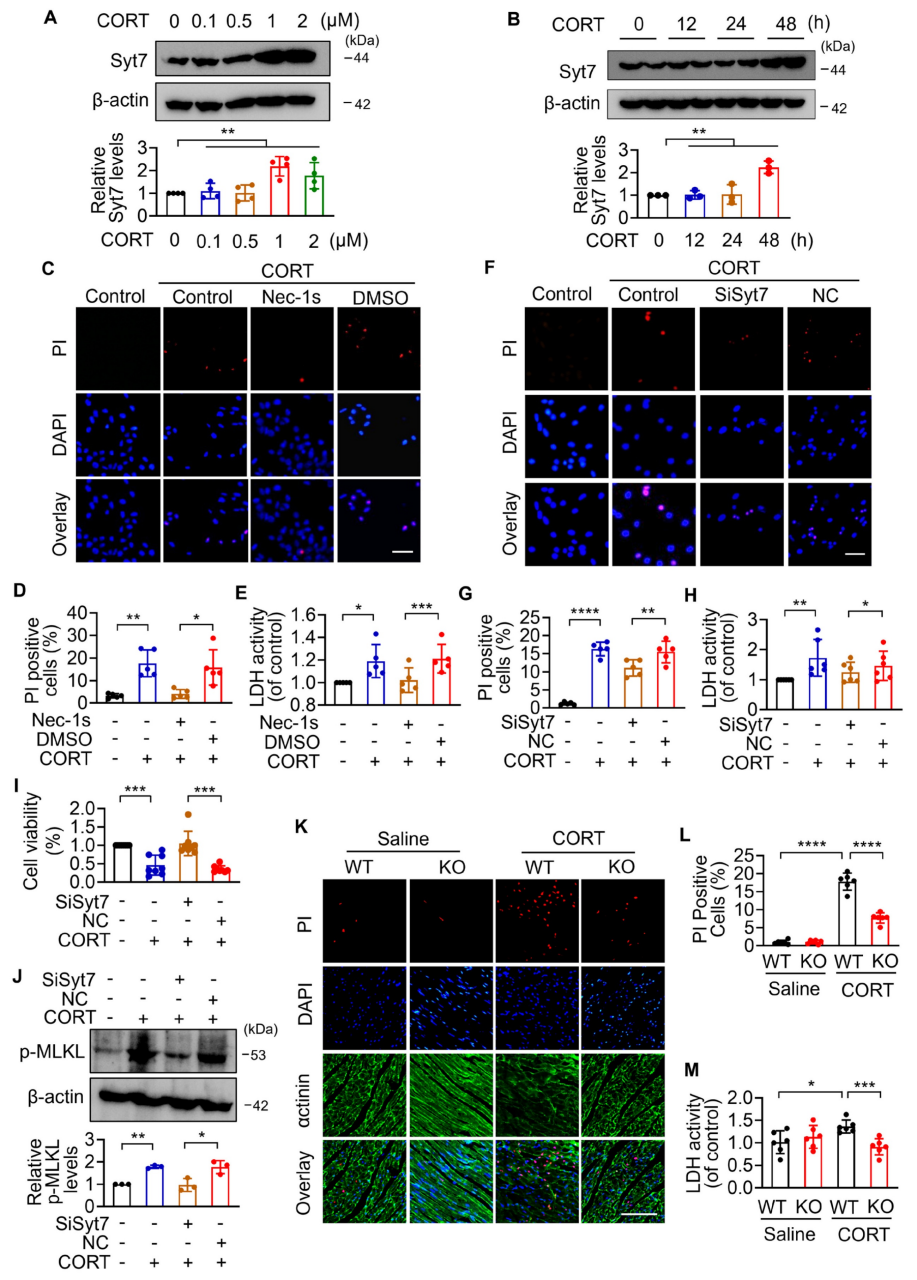
***Syt7* deficiency inhibits stress-induced necroptosis in cardiomyocytes and mouse hearts. A-B.** Syt7 protein levels in H9c2 cells under CORT treatment detected by Western blotting. ** p < 0.01. n = 4 (A). n = 3 (B). **C-E.** H9c2 cells were exposed to 50 µM Nec-1s and 1 µM CORT for 48 h. **C-D.** Propidium iodide (PI) staining. Bar = 50 µm. * p < 0.05. ** *p* < 0.01. n = 5.** E.** Lactate dehydrogenase (LDH) activity detection. * p < 0.05. *** *p* < 0.001. n = 5. **F-J.** H9c2 cells were infected with *Syt7* siRNA adenovirus, and then treated with 1 µM CORT for 48 h. **F-G.** Quantification of necrotic cells by PI staining. Bar = 50 µm. ** *p* < 0.01. **** *p* < 0.0001. n = 5. **H.** LDH activity detection. * p < 0.05. ** *p* < 0.01. n = 6. **I.** Cell viability analysis. *** *p* < 0.001. n = 8. **J.** Phosphorylated-MLKL (p-MLKL) levels detection. * p < 0.05. ** *p* < 0.01. n = 3. **K-M.** Analysis of cardiac necrosis in wild-type (WT) and *Syt7* knockout (KO) mice administered CORT. **K-L.** Cardiac necrosis detected by PI staining. Red: PI; blue: DAPI; green: α-actinin. Bar = 100 µm. **** *p* < 0.0001. n = 6. **M.** LDH activity detection. * p < 0.05. *** *p* < 0.001. n = 6. Data are expressed as mean ± standard deviation (SD).

To investigate the function of Syt7 in cardiac stress injury and the precise mechanisms, *in vitro* investigations were performed. The expression levels of Syt7 are markedly increased by CORT at a dose of 1µM (Figure 2A). Upon 1 µM CORT treatment, Syt7 increased in a time-dependent manner, with a significant elevation at 48 h (Figure 2B). Given the critical contribution of cell death to stress-induced cardiac disorders 15, we next attempted to study the ways involved in CORT-induced cardiomyocyte death. Apoptosis, a well-established programmed cell death governed by caspases— such as caspase-3, -7, and -9 17, 46 — was firstly assessed. Under CORT treatment, the expression levels of cleaved caspase-9 and cleaved caspase-7 did not change significantly, while cleaved caspase-3 activity was suppressed by CORT (Figure S5A, S6A-C), suggesting that classical apoptotic pathways were not predominantly activated in this context. It was reported that inhibition of apoptosis probably induces necroptosis as a compensatory form of regulated cell death 47. We found that CORT significantly increased the PI-positive cells and the release of lactic dehydrogenase (LDH), both indicative of membrane rupture associated with necrotic cell death, which was notably attenuated by Nec-1s, a necroptosis inhibitor (Figure 2C-E). Similarly, *Syt7* knockdown markedly attenuated CORT-induced necroptosis, as evidenced by decreased proportion of PI-positive cells (Figure 2F-G), suppressed LDH activity (Figure 2H), enhanced cell viability (Figure 2I), and down-regulated phosphorylated-MLKL protein levels (Figure 2J). Consistent with these results, both *Syt7* knockout mice and cardiac-specific *Syt7* knockdown mice exhibited a significantly decreased necrotic cells and serum LDH activity compared to the control group (Figure 2K-M, S4I-J). Furthermore, enforced expression of *Syt7* markedly increased the sensitivity of cardiomyocytes to CORT-induced necroptosis, as demonstrated by a higher proportion of PI-positive cardiomyocytes, enhanced LDH activity, and reduced cell viability (Figure S5B, S7A-D). These effects were significantly attenuated by either Nec-1s (Figure S7E-G) or mitochondrial permeability transition pore (mPTP) inhibitor CsA (Figure S7H-J). In addition, we evaluated the potential involvement of ferroptosis and pyroptosis in stress-induced cardiomyocyte injury. It was shown that treatment with 1 μM CORT for 48 h did not induce significant activation of these cell death ways, and Syt7 did not participate in their regulation (Figure S6D-H). Collectively, these results indicate that Syt7 facilitates stress-induced myocardial injury mainly through regulating necroptosis.

### Synaptotagmin-7 regulates mitochondrial structural and metabolic dysfunction under stress

Necroptosis is regulated primarily through two major pathways: death receptor-induced necrosis and mitochondrial permeability transition pore (mPTP)-mediated necrosis 48. To determine whether Synaptotagmin-7 (Syt7) is involved in classical death receptor-dependent necroptosis, we employed a well-established model using TNF-α in combination with the pan-caspase inhibitor z-VAD-fmk 49. Notably, silencing of *Syt7* did not affect the extent of necrotic cell death in this model, as reflected by unchanged levels of necrotic cell death rate (Figure S8A) and LDH release (Figure S8B), suggesting that Syt7 is dispensable for death receptor-triggered necroptosis.

**Figure 3:**
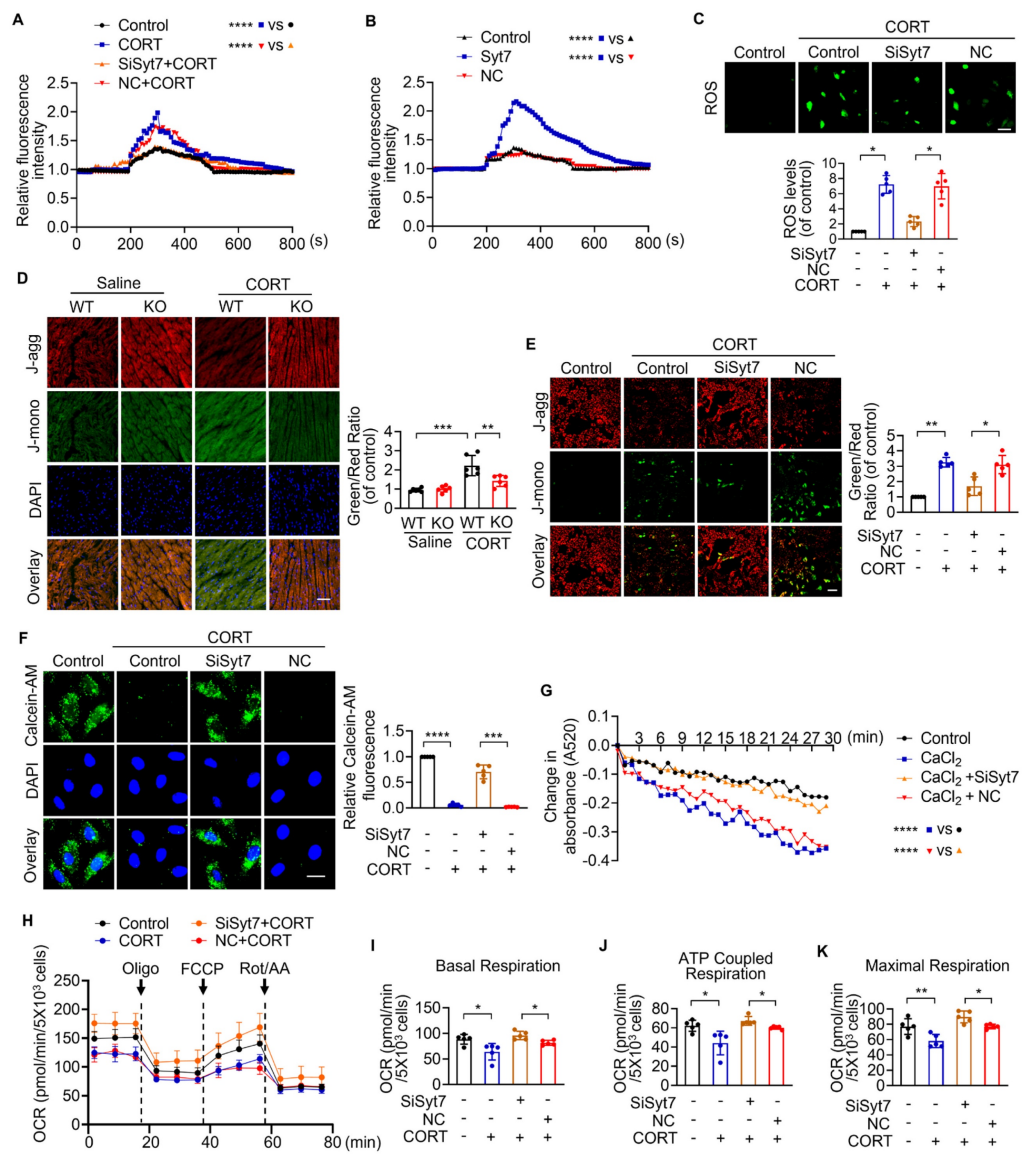
** Syt7 promotes necroptosis through regulation of mPTP opening and mitochondrial function. A-B.** Real-time monitoring of intracellular Ca²⁺ fluorescence intensity in H9c2 cells. KCl (50 mM) was added at 200 s. **** *p* < 0.0001. n = 5. **C.** Reactive oxygen species (ROS) levels detected in H9c2 cells using DCFH-DA probe. Bar = 100 µm. * *p* < 0.05. n = 5. **D-E.** Mitochondrial membrane potential (ΔΨm) assessed by 5,5',6,6'-tetrachloro-1,1',3,3'-tetraethyl-1H-imidazole-2-carboxylic acid (JC-1) in hearts (D, n = 6) and H9c2 cells (E, n = 5), respectively. Ratio of J-monomers to J-aggregates calculated. Bar = 50 µm. * *p* < 0.05. ** p < 0.01. *** *p* < 0.001. **F.** Mitochondrial permeability transition pore (mPTP) opening assessed in H9c2 cells by Calcein-AM staining. Bar = 20 µm. *** *p* < 0.001. **** *p* < 0.0001. n = 5. **G.** Mitochondrial swelling detected by absorbance over 30 min. **** *p* < 0.0001. n = 5. **H-K.** Real-time oxygen consumption rate (OCR) analysis in H9c2 cells. Basal, ATP-coupled, and maximal respiration rates were calculated. * *p* < 0.05. ** p < 0.01. n = 5. Data are expressed as mean ± standard deviation (SD).

To further investigate the function of Syt7 in mPTP-mediated necroptosis, we evaluated key upstream driven factors of mPTP opening, including particularly Ca²⁺ overload and oxidative imbalance 50. CORT significantly increased intracellular Ca²⁺ levels, which was markedly attenuated by *Syt7* knockdown (Figure 3A). Overexpression of *Syt7* further enhanced Ca²⁺ accumulation (Figure 3B). Additionally, CORT induced a substantial ROS accumulation in cardiomyocytes, which was suppressed upon *Syt7* silencing (Figure 3C). Given that both Ca²⁺ overload and ROS toxicity are potent activators of mPTP opening, the mitochondrial ΔΨm was analyzed using the fluorescent probe JC-1, a standard tool for assessing mitochondrial polarization. CORT led to a notable reduction in red J aggregates and a production in green J monomers, indicating ΔΨm dissipation. These effects were significantly mitigated in *Syt7* KO hearts (Figure 3D), Syt7 cardiac-specific knockdown hearts (Figure S4K) and *Syt7* knockdown cardiomyocytes (Figure 3E), suggesting that loss of Syt7 stabilizes mitochondrial membrane potential under stress. To directly analyze mPTP opening, calcein-AM staining assay and mitochondrial swelling assay were performed. Inhibition of Syt7 significantly alleviated CORT-induced mPTP opening, as evidenced by preserved calcein fluorescence (Figure 3F) and reduced CaCl₂-induced mitochondrial swelling (Figure 3G). Moreover, extracellular flux analysis revealed that *Syt7* silencing restored mitochondrial respiratory function in cardiomyocytes exposed to CORT. Specifically, knockdown of *Syt7* rescued basal respiration, ATP-linked respiration, and maximal respiratory capacity (Figure 3H-K). Additionally, reverse validation experiments demonstrated that *Syt7* overexpression exacerbated CORT-induced ROS accumulation, ΔΨm dissipation and mPTP opening (Figure S9A-C). The mPTP inhibitor CsA effectively attenuated Syt7-mediated ROS accumulation (Figure S9D). Taken together, these findings demonstrate that Syt7 regulates mPTP-mediated necroptosis through targeting Ca²⁺ overload, ROS accumulation and mitochondrial bioenergetics.

### The p53-Bak signaling axis drives mPTP opening and cardiomyocyte necroptosis

**Figure 4:**
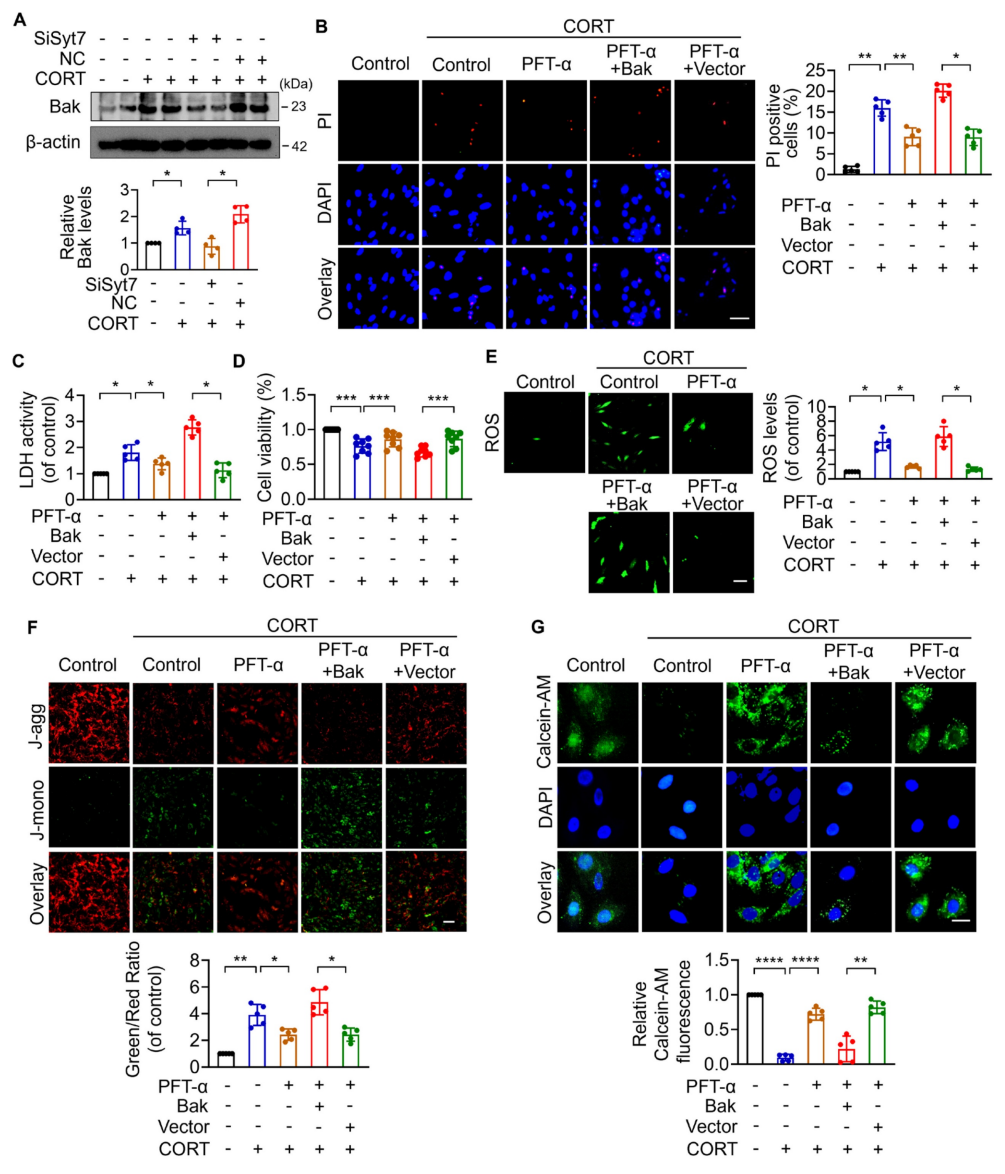
** The p53-Bak axis contributes to necroptosis and mPTP opening in cardiomyocytes. A.** Bcl-2 homologous antagonist/killer (Bak) protein levels detected by Western blotting after Si*Syt7* or NC treatment under CORT exposure. * *p* < 0.05. n = 4. **B-G.** H9c2 cells were exposed to PFT-α (a specific inhibitor of p53) with transfected with either a *Bak* overexpression plasmid (pcDNA3.1 vector containing *Bak* cDNA) or the empty pcDNA3.1 vector, followed by CORT treatment. **B.** PI staining analysis. Bar = 50 µm. * p < 0.05. ** p < 0.01. n = 5. **C.** LDH activity assay. * *p* < 0.05. n = 5. **D.** Cell viability detection. *** *p* < 0.001. n = 8. **E.** ROS levels detected by the fluorescent probe DCFH-DA. Bar = 100 µm. * *p* < 0.05. n = 5. **F.** ΔΨm detected by JC-1 staining. The ratio of J-monomers to J-aggregates was calculated. Bar = 50 µm. * p < 0.05. ** *p* < 0.01. n = 5. **G.** Assessment of mPTP opening by Calcein-AM fluorescence staining. Bar = 20 µm. ** p < 0.01. **** *p* < 0.0001. n = 5. Data are expressed as mean ± standard deviation (SD).

Next, we explored the downstream targets of Syt7 in the regulation of stress-induced necroptosis and mitochondrial dysfunction. The Bcl-2 protein family, including Bax, Bak, Bim, and Bik, was analyzed due to their pivotal roles in regulating outer mitochondrial membrane integrity and mPTP opening 51-55. Results showed that silencing of *Syt7* did not significantly affect the expression levels of Bax, Bim, or Bik (Figure S10A-C), while it significantly attenuated CORT-upregulated Bak expression (Figure 4A), suggesting that Bak is a potential downstream target of Syt7 in the stress pathway. Subcellular localization analysis in cardiomyocytes revealed that Syt7 is broadly distributed in the cytoplasm, mitochondria, and nucleus, with a predominant accumulation in the nucleus (Figure S11). Therefore, we speculated that Syt7 regulated stress-induced injury probably through modulating nuclear processes such as transcription. Given that *Bak* is a well-established transcriptional target of p53 56, 57, and that p53 has been involved in regulating mPTP opening across various pathological contexts including cardiac ischemia/reperfusion (I/R) injury and oxidative stress in H9c2 cells 48, NaF-induced neurotoxicity and PC12 cell apoptosis 58, as well as T-2 toxin-induced cartilage degeneration in Kashin-Beck disease models and ATDC5 cell apoptosis 59, we explored whether p53-Bak axis contribute to stress-induced cardiomyocyte necrosis. Pifithrin-α (PFT-α), a specific inhibitor of p53, significantly reduced CORT-induced necroptosis, as evidenced by reduced PI-positive cell, decreased LDH activity and enhanced cell viability, which was attenuated by *Bak* overexpression (Figure 4B-D). Furthermore, *Bak* overexpression attenuated the inhibitory effects of PFT-α on mitochondrial dysfunction including ROS accumulation, ΔΨm dissipation, and mPTP abnormal opening (Figure 4E-G). These findings indicate that p53 and Bak constitute a regulatory axis in necroptosis and mitochondrial dysfunction under stress.

### Synaptotagmin-7 interacts with p53 in stress-induced necrotic signaling

**Figure 5:**
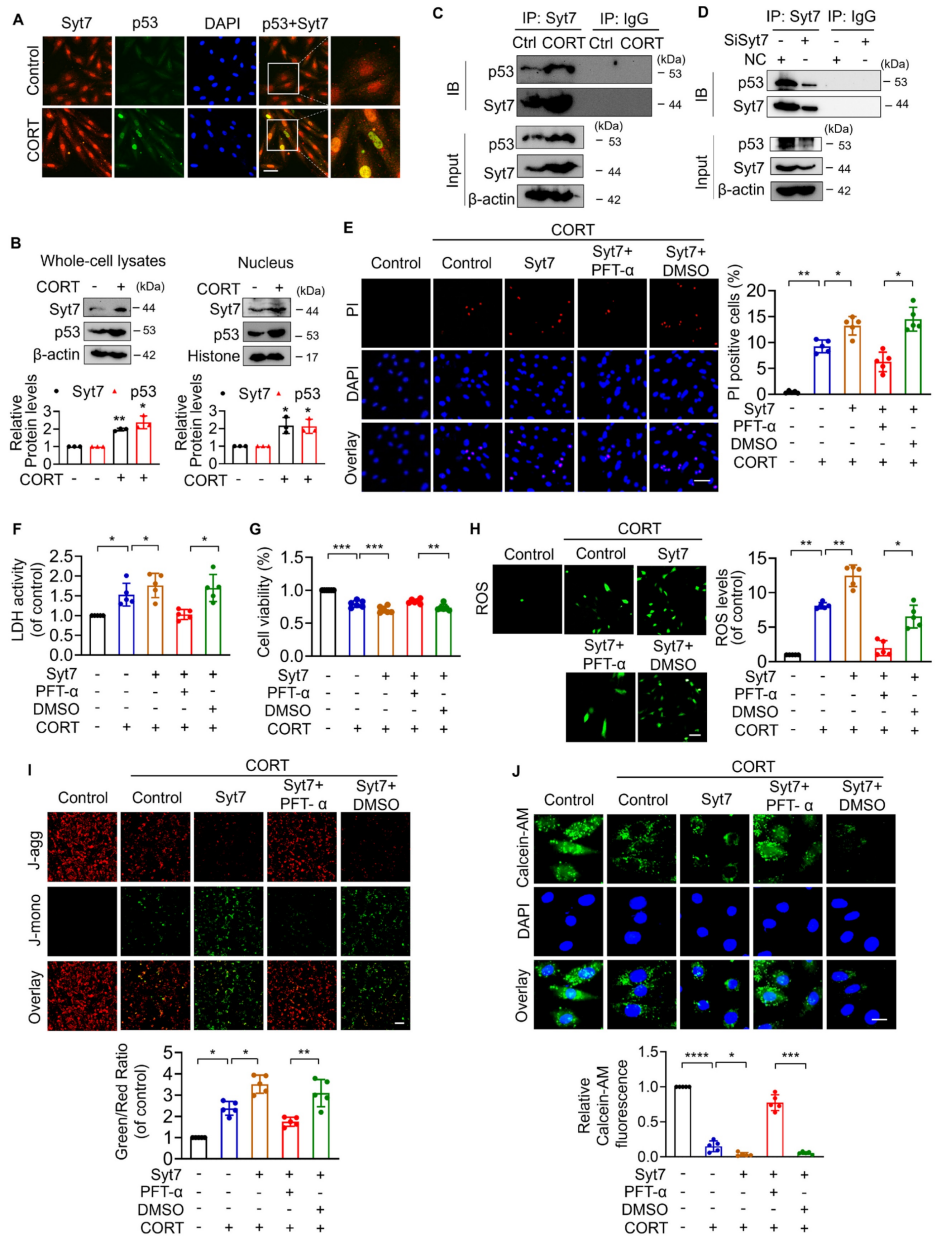
** Syt7 interacts with p53 to promote mPTP opening and necroptosis. A.** Immunofluorescence staining for Syt7 and p53 in H9c2 cells after 48 h of CORT treatment. Red: Syt7; green: p53; blue: DAPI. Bar = 50 µm. **B.** Western blot analysis of Syt7 and p53 expression levels in whole-cell and nuclear fractions under CORT treatment. * *p* < 0.05. ** *p* < 0.01. n = 3. **C.** Co-immunoprecipitation (co-IP) of Syt7 and p53 in H9c2 cells exposed to CORT for 48 h.** D.** Co-IP analysis of Syt7 and p53 interaction following *Syt7* siRNAs treatment. **E-J.** H9c2 cells were transfected with *Syt7* overexpression plasmid with or without PFT-α (a specific inhibitor of p53), followed by CORT treatment. **E.** PI staining. Bar = 50 µm. * *p* < 0.05. ** p < 0.01. n = 5. **F.** LDH activity detection. * *p* < 0.05. n = 5. **G.** Cell viability detection. ** *p* < 0.01. *** *p* < 0.001. n = 6. **H.** Intracellular ROS levels analysis by the fluorescent probe DCFH-DA. Bar = 100 µm. * *p* < 0.05. ** *p* < 0.01. n = 5. **I.** ΔΨm detected by JC-1 staining. The ratio of J-monomers to J-aggregates was calculated. Bar = 50 µm. * *p* < 0.05. ** *p* < 0.01. n = 5.** J.** The mPTP opening evaluated by Calcein-AM fluorescence staining. Bar = 20 µm. * *p* < 0.05. *** *p* < 0.001. **** *p* < 0.0001. n = 5. Data are expressed as mean ± standard deviation (SD).

To further study the molecular mechanism underlying Syt7-mediated necroptosis, the interaction between Syt7 and p53 was explored. Immunofluorescence analysis revealed that Syt7 was predominantly localized in the cytoplasm and nucleus, while p53 expression was relatively low and primarily cytoplasmic, under physiological conditions. After CORT treatment, both Syt7 and p53 were upregulated and exhibited increased nuclear co-localization (Figure 5A). Consistently, subcellular fractionation confirmed that CORT induced nuclear translocation and accumulation of both proteins (Figure 5B). Furthermore, co-immunoprecipitation (co-IP) assay was performed in cardiomyocytes to determine whether Syt7 interacts with p53. CORT markedly promoted the interaction between Syt7 and p53 (Figure 5C), whereas *Syt7* knockdown markedly reduced this interaction (Figure 5D). We wonder whether Syt7 regulates necrosis by targeting p53. Overexpression of *Syt7* significantly increased CORT-induced necrotic cell death, LDH release and decreased cell viability, which were attenuated by p53 inhibitor PFT-α (Figure 5E-G). In addition, *Syt7* overexpression exacerbated CORT-induced mitochondrial dysfunction, including elevated ROS accumulation, mitochondrial ΔΨm dissipation, and prolonged mPTP opening. Inhibition of p53 significantly mitigated these mitochondrial impairments (Figure 5H-J). Taken together, these findings suggest that Syt7 interacts with p53 to promote mPTP opening and mitochondrial necroptosis.

### Syt7 enhances p53-dependent Bak transcription to promote mitochondrial necroptosis

**Figure 6:**
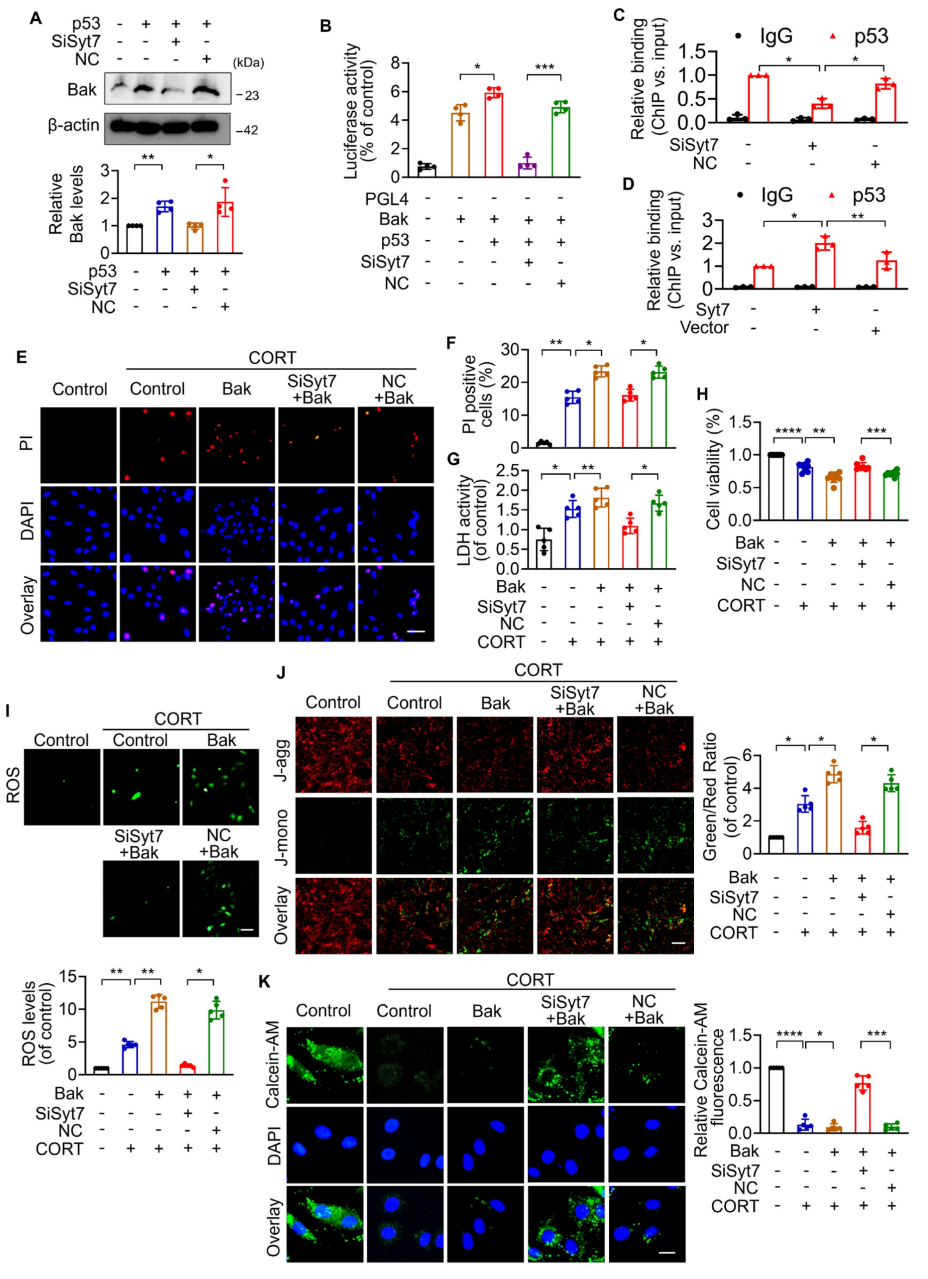
** Syt7 regulates mPTP opening and necroptosis through the p53-Bak axis. A.** Western blot analysis of Bak expression following *p53* overexpression with or without *Syt7* siRNAs treatment. * p < 0.05. ** p < 0.01. n = 4. **B.** Dual-luciferase assay of *Bak* promoter activity after co-transfection of *p53* plasmid with or without *Syt7* siRNAs in HEK293 cells. * *p* < 0.05. *** p < 0.001. n = 4.** C-D.** Chromatin immunoprecipitation quantitative PCR (ChIP-qPCR) to assess p53 binding to the *Bak* promoter under *Syt7* siRNA (C) or *Syt7* overexpression plasmid (D) in H9c2 cells. IgG used as control. * *p* < 0.05. ** p < 0.01. n = 3. **E-K.** H9c2 cells were transfected with *Bak* plasmid with or without *Syt7* siRNA, followed by CORT treatment. **E-F.** PI staining assay. Bar = 50 µm. * *p* < 0.05. ** p < 0.01. n = 5. **G.** LDH activity detection. * *p* < 0.05. ** p < 0.01. n = 5. **H.** Cell viability analysis. ** *p* < 0.01. *** p < 0.001. **** p < 0.0001. n = 8. **I.** ROS levels detected by the fluorescent probe DCFH-DA. Bar = 100 µm. * p < 0.05. ** p < 0.01. n = 5. **J.** ΔΨm detected by JC-1 staining, calculated as the ratio of J-monomers to J-aggregates. Bar = 50 µm. * p < 0.05. n = 5. **K.** mPTP opening was evaluated using Calcein-AM staining. Bar = 20 µm. * *p* < 0.05. *** *p* < 0.001. **** p < 0.0001. n = 5. Data are expressed as mean ± standard deviation (SD).

We next investigated whether Synaptotagmin-7 (Syt7) facilitates the transcriptional activation of *Bak* by p53, thereby contributing to mPTP opening as well as necroptosis. As shown in our previous results, corticosterone (CORT) treatment significantly upregulated Bak expression in cardiomyocytes, an effect that was markedly attenuated by *Syt7* knockdown (Figure 4A). Overexpression of *p53* significantly up-regulated Bak, whereas *Syt7* silencing diminished p53-induced Bak upregulation (Figure 6A). To determine the underlying mechanism, a *Bak* promoter fragment containing a putative *p53* binding site was cloned into a firefly luciferase reporter vector (pGL4). Dual-Luciferase reporter assays in cardiomyocytes showed that the elevated luciferase activity in presence of *Bak* promoter construct was further enhanced by co-transfection with the *p53* overexpression plasmid, whereas *Syt7* knockdown significantly suppressed the chemiluminescence signal (Figure 6B), indicating that Syt7 modulates p53-driven *Bak* promoter activation. Chromatin immunoprecipitation (ChIP)-PCR further confirmed the direct binding of p53 to the *Bak* promoter in cardiomyocytes. Notably, *Syt7* silencing reduced, while *Syt7* overexpression enhanced, the enrichment of p53 on the *Bak* promoter (Figure 6C-D), suggesting that Syt7 strengthens the transcriptional interaction between p53 and *Bak*. Functionally, *Bak* overexpression exacerbated CORT-induced cardiomyocyte necrosis and mitochondrial dysfunction, which was significantly attenuated by* Syt7* knockdown (Figure 6E-K). Collectively, these results demonstrate that Syt7 regulates mitochondrial necroptosis through facilitating p53-mediated transcription of Bak.

### Therapeutic silencing of Synaptotagmin-7 protects against stress-induced cardiac injury

**Figure 7:**
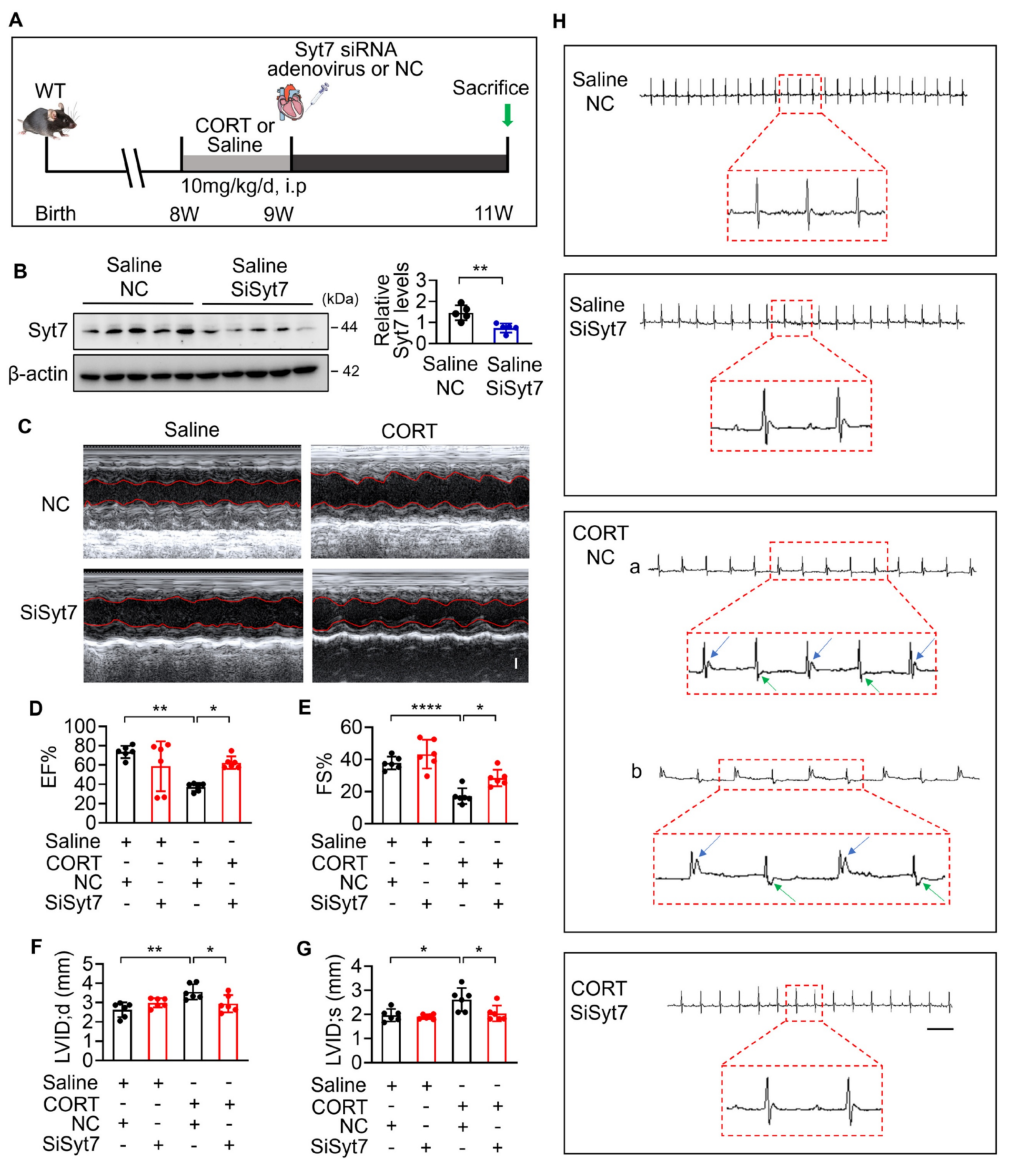
***Syt7* silencing mitigates CORT-induced cardiac structural and electrical remodeling. A.** Schematic diagram of the *in vivo* experimental protocol involving *Syt7* siRNA adenovirus administration following CORT treatment. **B.** Western blot analysis of Syt7 protein expression. ** *p* < 0.01. n = 5. **C-G.** Representative echocardiograms (C) and quantitative analysis of cardiac functional parameters (D-G), including EF, FS, LVID;d, and LVID;s. Bar = 1 mm. * *p* < 0.05. ** *p* < 0.01. **** *p* < 0.0001. n = 6. **H.** Representative electrocardiograms. Bar = 500 ms. Data are expressed as mean ± standard deviation (SD).

**Figure 8:**
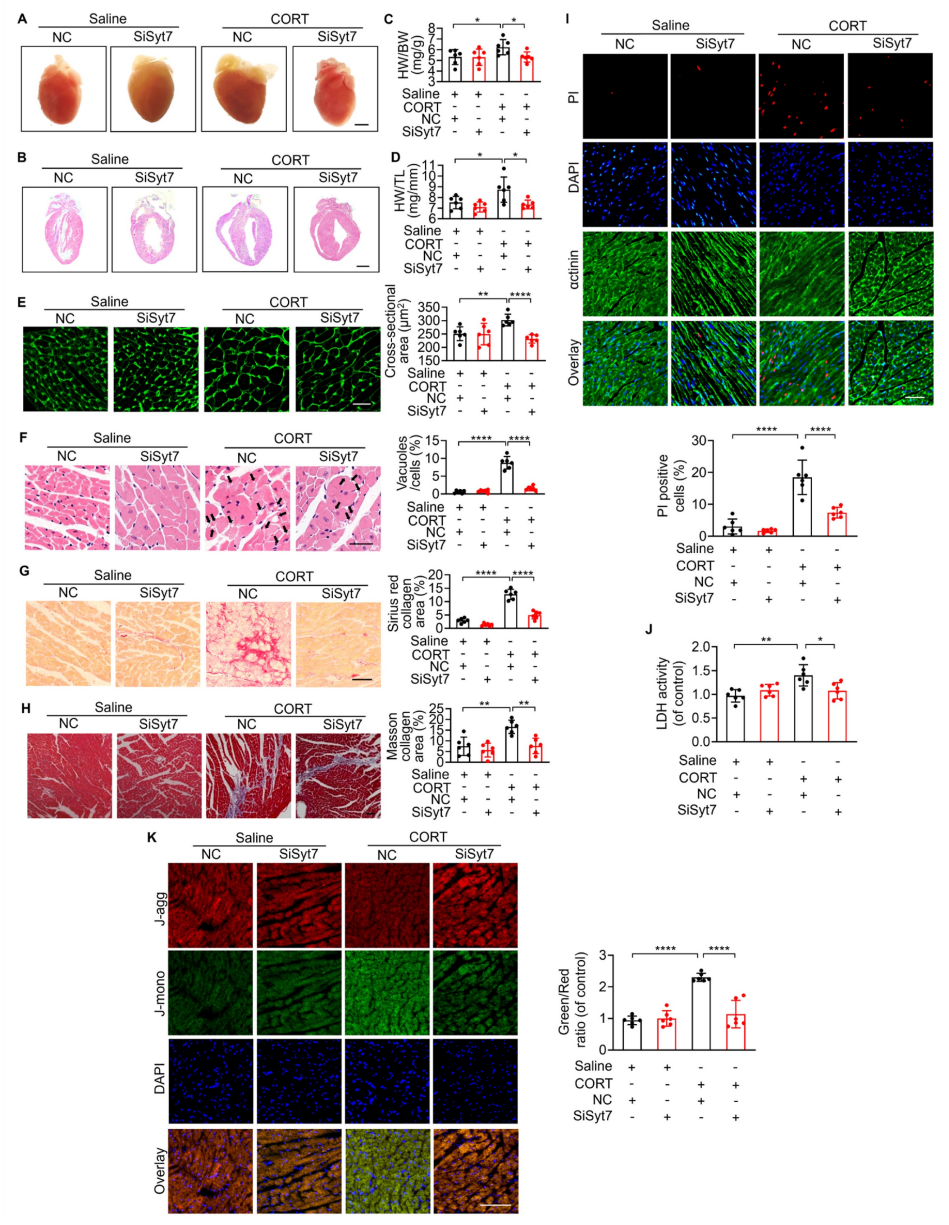
** Syt7 inhibition attenuates CORT-induced myocardial injury and necroptosis. A-B.** Gross heart images and H&E-stained longitudinal sections. Bar = 2 mm. **C-D.** Heart weight to body weight ratio (HW/BW, mg/g) and heart weight to tibia length ratio (HW/TL, mg/mm). * p < 0.05. n = 6. **E.** Cross-sectional area of cardiomyocytes assessed by WGA staining. Bar = 30 µm. ** p < 0.01. **** *p* < 0.0001. n = 6. **F.** Vacuolar degeneration in cardiomyocytes detected by H&E staining. Arrows indicate vacuolar degeneration. Bar = 30 µm. **** p < 0.0001. n = 6. **G.** Myocardial fibrosis evaluated by Sirius Red staining. Bar = 50 µm. **** *p* < 0.0001. n = 6. **H.** Myocardial fibrosis assessed using Masson's trichrome staining. Bar = 50 µm. ** *p* < 0.01. n = 6. **I.** Cardiomyocyte necrosis detected by PI staining. Red: PI; blue: DAPI; green: α-actinin. Bar = 50 µm. **** *p* < 0.0001. n = 6. **J.** LDH activity. * *p* < 0.05. ** *p* < 0.01. n = 6. **K.** ΔΨm assessed by JC-1 staining, calculated as the J-monomers to J-aggregates ratio. Bar = 100 µm. **** *p* < 0.0001. n = 6. Data are expressed as mean ± standard deviation (SD).

While our study has elucidated key mechanistic insights into stress-related myocardial injury, clinical manifestations remain heterogeneous, and effective therapeutic strategies are still lacking 12. To evaluate the translational potential of Syt7, we investigated whether *Syt7*-targeted siRNA therapy could mitigate CORT-induced cardiac injury. WT mice received intraperitoneal injection with saline or CORT, followed by *in situ* myocardial injection of either *Syt7*-targeting siRNA adenovirus or a negative control vector (Figure 7A). Adenoviral *Syt7* siRNA significantly suppressed Syt7 expression in myocardial tissue (Figure 7B), without affecting its expression in non-cardiac tissues including the liver, spleen, lung, kidney, and brain (Figure S12). Mice from *Syt7* siRNA group exhibited preserved cardiac function including enhanced EF, improved FS, and decreased LVID;d and LVID;s (Figure 7C-G), and mitigated T-wave alternans (Figure 7H), compared with the negative control group. Inhibition of Syt7 reduced myocardial hypertrophy, as evidenced by reduced heart volume (Figure 8A-B), decreased ratio of HW/BW and HW/TL (Figure 8C-D), and smaller cardiomyocyte cross-sectional areas (Figure 8E). Diminished myocardial vacuolar degeneration (Figure 8F) and attenuated interstitial fibrosis (Figure 8G-H) were observed in the *Syt7* siRNA group. Furthermore, *Syt7* knockdown reduced CORT-induced cardiomyocyte necrosis (Figure 8I-J) and mitochondrial injury (Figure 8K). Taken together, these findings demonstrate targeting *Syt7* with siRNA adenoviral therapy alleviates cardiac dysfunction, remodeling, fibrosis, and mitochondrial-mediated necrosis.

## Discussion

Stress-induced cardiac injury is characterized by a complex interplay of adaptive and maladaptive responses. Although acute stress may temporarily preserve cardiovascular homeostasis, chronic or excessive stress can result in progressive myocardial damage and functional decline 60. Among various types of programmed cell death implicated in cardiac damage, necroptosis has gained increasing attention. However, the precise mechanisms are not yet fully clarified. The present work identifies Syt7 as a novel regulator of stress-induced cardiac injury. Syt7 expression is significantly upregulated in both stress-threatened hearts and cardiomyocytes. *Syt7* deficiency markedly ameliorated CORT-induced cardiac dysfunction, arrhythmia, remodeling, fibrosis and necroptosis. Mechanistically, Syt7 interacts with p53, facilitates p53-directed transcription of Bak, thereby promoting mitochondrial dysfunction and necroptosis. Importantly, *Syt7*-targeting siRNA adenoviral therapy mitigates stress-induced cardiomyocyte necrosis and restores cardiac performance. These findings collectively establish Syt7 as a critical driver of stress-induced necroptosis and a promising therapeutic target.

Chronic exposure to stress activates the hypothalamic-pituitary-adrenal (HPA) axis, thereby increasing secretion of CORT in rodents 61, 62. Circulating CORT content in stressed mice typically range from 300 to 600 ng/mL 15, 37, 63. 1 μM CORT has been widely used to simulate glucocorticoid-induced stress* in vitro*. Previous studies have shown that 1 μM CORT impairs cardiomyocyte viability and contractile function 15, enhances L-type calcium currents in ventricular myocytes 64, and triggers mitophagy in hippocampal neurons 37. It also affects neural stem cell differentiation and promotes Aβ generation in neuroblastoma cells 65, 66. In our study, a CORT concentration gradient was established to assess its regulatory effects on Syt7 expression in cardiomyocytes and it was shown that 1 μM CORT significantly upregulated Syt7 protein levels (Figure 2A), suggesting a potential threshold for stress-induced Syt7 activation. Based on these observations, we employed 10 mg/kg CORT *in vivo* and 1 μM CORT *in vitro* to model pathologically relevant stress conditions. The type of cell death triggered by stress is highly context-dependent, varying with CORT concentration, exposure duration, and cell type. Apoptosis has been widely reported in neuronal cells exposed to 200-400 μM CORT for 24-48 h, as evidenced by caspase activation and chromatin condensation in PC12 and HT22 cells 67-71. Other types of programmed cell death, such as ferroptosis and pyroptosis, are activated under prolonged or combinatorial stress conditions—particularly with long-term CORT administration or co-stimulation with lipopolysaccharide (LPS) 72-75. Autophagy is also frequently activated in response to stress. Models employing chronic restraint or sustained CORT exposure demonstrate increased autophagic flux in both neuronal and cardiac tissues 63, 76. More specifically, exposure to 1 μM CORT has been shown to induce autophagy in neuronal cells within 24 h 37, and in cardiomyocytes, short-term CORT treatment (6 h) leads to mitochondrial dysfunction, superoxide (O₂⁻) accumulation, inflammatory activation, lipid peroxidation, and ferroptosis 15. Similarly, in hippocampal neural stem/progenitor cells, 48 h exposure to 1 μM CORT results in glutathione (GSH) depletion, increased ROS levels, elevated lipid peroxidation, and suppression of the LC3-II/I ratio, confirming concurrent activation of ferroptotic and autophagic pathways 75. However, despite growing attention in these stress-induced cell death pathways, the involvement of necroptosis in CORT-induced cardiac injury has remained largely unexplored 77. Our study has demonstrated that 1 μM CORT treatment for 48 h induces prominent necroptotic features in cardiomyocytes, including increased PI uptake, elevated LDH release, reduced cell viability, intracellular Ca²⁺ overload, ROS accumulation, and mPTP prolonged opening. Notably, inhibition of *Syt7* significantly attenuated these pathological changes, highlighting its essential role in mediating stress-induced necroptosis. Interestingly, in the CORT-induced cardiomyocyte injury model established in our study, other programmed cell death including apoptosis, ferroptosis, and pyroptosis did not exhibit significant activation. However, this observation should be interpreted with caution, as the assessment was limited to a select subset of representative markers, and may not comprehensively capture the full spectrum of cell death responses. It is needed to confirm whether alternative modes of cell death may also contribute and the possible crosstalk with necroptosis in the future.

Syt7 has been identified as a calcium sensor originally for its role in vesicle trafficking, exocytosis, and neurotransmitter release 78, 79. Beyond its neuronal functions, Syt7 mediates hormonal secretion and participates in development and progression of multiple cancers, gastric, hepatic, pulmonary, thyroid, ovarian, cervical, and hematologic malignancies 28-31, 80-83. In contrast, studies investigating Syt7 in cardiovascular biology remain limited. Syt7 is highly enriched at the sympathetic nerve terminals of the mouse heart, where it modulates neurotransmission through a calcium-independent mechanism. *Syt7* knock-in mice exhibit enhanced norepinephrine (NE) release and elevate systemic blood pressure, which indicates a role in autonomic regulation of cardiovascular function 33. Epigenetic profiling has revealed that hypertension development has been closely related to methylation of the *Syt7* promoter 34. Recent evidence has also shown that silencing *Syt7* in cardiomyocytes inhibits hypoxia/reoxygenation (H/R)-induced RIP3 upregulation and LDH activity elevation 84. These findings collectively support a multifaceted role for Syt7 in cardiovascular pathology, including sympathetic neurotransmission modulation, epigenetic control, and cellular stress responses. In addition, our previous work demonstrated that Syt7 is significantly increased upon Angiotensin II treatment and promoted cardiac hypertrophy by regulating autophagy 36. In the present study, we extend this understanding by providing the direct evidence that Syt7 functions as a critical mediator of stress-induced necroptosis and cardiac injury. Mechanistically, Syt7 regulates CORT-induced intracellular calcium overload and ROS accumulation—two well-established triggers of mPTP opening. mPTP prolonged opening leads to mitochondrial swelling, membrane rupture, and cell necroptosis, representing a key pathway of intrinsic regulated necroptosis. We have additionally assessed whether Syt7 is involved in the signaling of extrinsic necroptosis. Functional analysis revealed that *Syt7* knockdown did not significantly alter necrotic phenotypes, suggesting that Syt7 is dispensable for extrinsic necrosis. From a therapeutic perspective, silencing *Syt7* markedly reduced these mitochondrial insults and conferred significant protection against myocardial injury. Together, these results position Syt7 as a central regulator of calcium signaling, redox stress, and regulated necrosis in the stressed heart.

A major advance of the present study is identification of p53 as a critical downstream target of Syt7 in stress-induced necrotic signaling. p53, a transcription factor, regulates diverse cellular stress signals to orchestrate apoptosis, autophagy, ferroptosis and necroptosis 85. p53 has been demonstrated to regulate RIP1/RIP3-mediated necrosis in myocardial infarction 86. Additionally, p53 activates necrosis in heat-stressed intestinal epithelium via MLKL signaling 87 and interacts with Cyclophilin D and Drp1 to modulate mitochondrial permeability transition 88. Our work has revealed that Syt7 facilitates transcriptional activity of p53, leading to upregulation of Bak at the transcriptional levels. ChIP and luciferase assays confirmed that Syt7 enhances p53 binding to the *Bak* promoter. Functional assays revealed that p53 inhibition reversed the necroptotic effects of Syt7, while *Bak* overexpression abolished the protective effects of p53 blockade. These results place Bak downstream of the Syt7-p53 axis and identify it as a key mediator of mPTP opening and necrosis. Although Bak has been classically viewed as a pro-apoptotic effector, increasing evidence implicates it in necroptosis through disruption of mitochondrial membrane integrity 53, 89, 90.* Bak* knockout has been shown to reduce necrosis in mouse embryonic fibroblasts (MEFs) and hepatocellular carcinoma cells, particularly in the context of p53 activation 91. Our study has demonstrated that the p53-Bak axis critically mediates mPTP-dependent necroptosis in stressed cardiomyocytes.

The demonstration that *Syt7*-targeted siRNA therapy mitigates stress-induced cardiac injury highlights the translational potential of this pathway. Adenoviral delivery of *Syt7* siRNA restored systolic function, improved ventricular remodeling, and reduced fibrosis and cellular necrosis. Given the lack of effective therapies for stress cardiomyopathy, targeting Syt7 may offer a new strategy for modulating stress responses in the heart. Several important questions remain. First, it is unclear whether Syt7 regulates additional p53 targets involved in mitochondrial networks. Second, p53 is modulated by a variety of post-translational events, especially acetylation and phosphorylation, which may influence its activity under stress 87. Investigating how Syt7 influences these modifications could yield deeper mechanistic insights. Third, whether cardiomyocyte-specific Syt7 inhibition confers protection in other models of cardiac stress, such as pressure overload-triggered hypertrophic remodeling or ischemia/reperfusion damage, to assess the generalizability of its therapeutic efficacy. Moreover, the development of small-molecule Syt7 inhibitors may represent a promising direction for translational advancement, potentially offering a more clinically feasible and scalable intervention compared to gene therapy approaches. Finally, potential crosstalk between necroptosis and other death programs such as ferroptosis and pyroptosis merits further exploration, particularly in chronic or multifactorial stress models.

## Conclusions

This study identifies Syt7 as a novel stress-responsive factor that promotes stress-induced cardiomyocyte necroptosis, progressive myocardial damage and functional decline. Mechanistically, Syt7 interacts with p53, enhances its transcription of *Bak*, and thereby triggers mitochondrial dysfunction and necrosis, revealing a novel signaling axis of Syt7-p53-Bak. *Syt7*-targeted therapy mitigates stress-induced cardiac disorders, providing key insight for clinical treatment.

## Supplementary Material

Supplementary figures.

## Abbreviations

Syt7: Synaptotagmin-7
CORT: corticosterone
mPTP: mitochondrial permeability transition pore
EF: ejection fraction
FS: fraction shortening
LVID;d: left ventricular internal diameter at diastole
LVID;s: left ventricular internal diameter at systole
LVEDV: left ventricular end-diastolic volume
LVESV: left ventricular end-systolic volume
H&E: hematoxylin and eosin
PI: propidium iodide
LDH: lactate dehydrogenase
DMEM: Dulbecco's Modified Eagle's Medium
ΔΨm: mitochondrial membrane potential
JC-1: 5,5',6,6'-tetrachloro-1,1',3,3'-tetraethylbenzimidazolylcarbocyanine iodide
ROS: reactive oxygen species
ChIP-qPCR: Chromatin immunoprecipitation quantitative PCR
HPA: hypothalamic-pituitary-adrenal
RIP1: receptor-interacting protein kinase 1
RIP3: receptor-interacting protein kinase 3
HW: heart weight
BW: body weight
TL: tibia length
Bax: Bcl-2 associated X protein
Bak: Bcl-2 homologous antagonist/killer
Bim: Bcl-2 interacting mediator of cell death
Bik: Bcl-2 interacting killer
TNF-α: tumor necrosis factor-α
MLKL: mixed lineage kinase domain-like
Drp1: dynamin-related protein 1
GSH: glutathione
WGA: wheat germ agglutinin
HRP: horseradish peroxidase
PFT-α: Pifithrin-α
IP: immunoprecipitation
OCR: oxygen consumption rate

## References

[1] McEwen BS (1998). Protective and damaging effects of stress mediators. N Engl J Med.

[2] Steptoe A, Kivimäki M (2012). Stress and cardiovascular disease. Nat Rev Cardiol.

[3] Framke E, Sørensen JK, Andersen PK, Svane-Petersen AC, Alexanderson K, Bonde JP (2020). Contribution of income and job strain to the association between education and cardiovascular disease in 1.6 million Danish employees. Eur Heart J.

[4] Batty GD, Hamer M, Gale CR (2021). Life course psychological distress and cardiovascular disease risk factors in middle age: birth cohort study. Cardiovasc Res.

[5] Münzel T, Gori T, Babisch W, Basner M (2014). Cardiovascular effects of environmental noise exposure. Eur Heart J.

[6] Cohen S, Murphy MLM, Prather AA (2019). Ten Surprising Facts About Stressful Life Events and Disease Risk. Annu Rev Psychol.

[7] Song H, Fang F, Arnberg FK, Mataix-Cols D, Fernández de la Cruz L, Almqvist C (2019). Stress related disorders and risk of cardiovascular disease: population based, sibling controlled cohort study. BMJ.

[8] Kwok MK, Kawachi I, Rehkopf D, Schooling CM (2020). The role of cortisol in ischemic heart disease, ischemic stroke, type 2 diabetes, and cardiovascular disease risk factors: a bi-directional Mendelian randomization study. BMC Med.

[9] Saban KL, Mathews HL, Bryant FB, Tell D, Joyce C, DeVon HA (2018). Perceived discrimination is associated with the inflammatory response to acute laboratory stress in women at risk for cardiovascular disease. Brain Behav Immun.

[10] Burroughs Peña MS, Mbassa RS, Slopen NB, Williams DR, Buring JE, Albert MA (2019). Cumulative Psychosocial Stress and Ideal Cardiovascular Health in Older Women. Circulation.

[11] Kivimäki M, Steptoe A (2018). Effects of stress on the development and progression of cardiovascular disease. Nat Rev Cardiol.

[12] Sara JDS, Toya T, Ahmad A, Clark MM, Gilliam WP, Lerman LO (2022). Mental Stress and Its Effects on Vascular Health. Mayo Clin Proc.

[13] Gianaros PJ, Jennings JR (2018). Host in the machine: A neurobiological perspective on psychological stress and cardiovascular disease. Am Psychol.

[14] Apaydin DC, Jaramillo PAM, Corradi L, Cosco F, Rathjen FG, Kammertoens T (2020). Early-Life Stress Regulates Cardiac Development through an IL-4-Glucocorticoid Signaling Balance. Cell Rep.

[15] Yin Z, Ding G, Chen X, Qin X, Xu H, Zeng B (2020). Beclin1 haploinsufficiency rescues low ambient temperature-induced cardiac remodeling and contractile dysfunction through inhibition of ferroptosis and mitochondrial injury. Metabolism.

[16] Zhang XH, Wu JX, Sha JZ, Yang B, Sun JR, Bao ED (2020). Heat shock protein 90 relieves heat stress damage of myocardial cells by regulating Akt and PKM2 signaling in vivo. Int J Mol Med.

[17] Yuan J, Ofengeim D (2024). A guide to cell death pathways. Nat Rev Mol Cell Biol.

[18] Yan ZY, Jiao HY, Chen JB, Zhang KW, Wang XH, Jiang YM (2021). Antidepressant Mechanism of Traditional Chinese Medicine Formula Xiaoyaosan in CUMS-Induced Depressed Mouse Model via RIPK1-RIPK3-MLKL Mediated Necroptosis Based on Network Pharmacology Analysis. Front Pharmacol.

[19] Qing W, Li F, Wang X, Quan C, Ouyang W, Liao Q (2018). Inhibiting RIP1 Improves Chronic Stress-Induced Cognitive Impairments in D-Galactose-Induced Aging Mice. Front Behav Neurosci.

[20] Zeb S, Ye H, Liu Y, Du HP, Guo Y, Zhu YM (2022). Necroptotic kinases are involved in the reduction of depression-induced astrocytes and fluoxetine's inhibitory effects on necroptotic kinases. Front Pharmacol.

[21] Bonora M, Giorgi C, Pinton P (2022). Molecular mechanisms and consequences of mitochondrial permeability transition. Nat Rev Mol Cell Biol.

[22] Bernardi P, Gerle C, Halestrap AP, Jonas EA, Karch J, Mnatsakanyan N (2023). Identity, structure, and function of the mitochondrial permeability transition pore: controversies, consensus, recent advances, and future directions. Cell Death Differ.

[23] Courtney KC, Mandal T, Mehta N, Wu L, Li Y, Das D (2023). Synaptotagmin-7 outperforms synaptotagmin-1 to promote the formation of large, stable fusion pores via robust membrane penetration. Nat Commun.

[24] Wu Z, Kusick GF, Berns MMM, Raychaudhuri S, Itoh K, Walter AM (2024). Synaptotagmin 7 docks synaptic vesicles to support facilitation and Doc2α-triggered asynchronous release. Elife.

[25] Ma Y, Guo J, Rao H, Xin J, Song X, Liu R (2024). The 8-oxoguanine DNA glycosylase-synaptotagmin 7 pathway increases extracellular vesicle release and promotes tumour metastasis during oxidative stress. J Extracell Vesicles.

[26] Wang QW, Lu SY, Liu YN, Chen Y, Wei H, Shen W (2020). Synaptotagmin-7 deficiency induces mania-like behavioral abnormalities through attenuating GluN2B activity. Proc Natl Acad Sci U S A.

[27] Xie Y, Zhi K, Meng X (2021). Effects and Mechanisms of Synaptotagmin-7 in the Hippocampus on Cognitive Impairment in Aging Mice. Mol Neurobiol.

[28] Zhang W, Long J, Tang P, Chen K, Guo G, Yu Z (2023). SYT7 regulates the progression of chronic lymphocytic leukemia through interacting and regulating KNTC1. Biomark Res.

[29] Liu X, Li R, Chen X, Yao J, Wang Q, Zhang J (2023). SYT7 is a key player in increasing exosome secretion and promoting angiogenesis in non-small-cell lung cancer. Cancer Lett.

[30] Kanda M, Tanaka H, Shimizu D, Miwa T, Umeda S, Tanaka C (2018). SYT7 acts as a driver of hepatic metastasis formation of gastric cancer cells. Oncogene.

[31] Dong S, Pan J, Shen YB, Zhu LX, Chen L, Zhu F (2022). SYT7 plays a role in promoting thyroid cancer by mediating HMGB3 ubiquitination. Endocr Relat Cancer.

[32] Fu Y, Tian G, Zhang Z, Yang X (2021). SYT7 acts as an oncogene and a potential therapeutic target and was regulated by ΔNp63α in HNSCC. Cancer Cell Int.

[33] Shih AM, Varghese L, Bittar A, Park SH, Chung JM, Shin OH (2016). Dysregulation of Norepinephrine Release in the Absence of Functional Synaptotagmin 7. J Cell Biochem.

[34] Zhang H, Wang A, Xu T, Mo X, Zhang Y (2021). Promoter DNA Methylation in GWAS-Identified Genes as Potential Functional Elements for Blood Pressure: An Observational and Mendelian Randomization Study. Front Genet.

[35] Xu W, Yao H, Wu Z, Yan X, Jiao Z, Liu Y (2024). Oncoprotein SET-associated transcription factor ZBTB11 triggers lung cancer metastasis. Nat Commun.

[36] Sun T, Han Y, Li JL, Wang S, Jing ZJ, Yan Z (2024). Synaptotagmin-7 mediates cardiac hypertrophy by targeting autophagy. FEBS J.

[37] Choi GE, Lee HJ, Chae CW, Cho JH, Jung YH, Kim JS (2021). BNIP3L/NIX-mediated mitophagy protects against glucocorticoid-induced synapse defects. Nat Commun.

[38] Sun T, Han Y, Li JL, Jiao XY, Zuo L, Wang J (2022). FOXO3a-dependent PARKIN negatively regulates cardiac hypertrophy by restoring mitophagy. Cell Biosci.

[39] Sun T, Li J, Wang S, Han Y, Tao X, Yuan M (2025). Synaptotagmin-1 attenuates myocardial programmed necrosis and ischemia/reperfusion injury through the mitochondrial pathway. Cell Death Dis.

[40] Tokuoka H, Goda Y (2003). Synaptotagmin in Ca2+ -dependent exocytosis: dynamic action in a flash. Neuron.

[41] Yu S, Zhao Y, Luo Q, Gu B, Wang X, Cheng J (2024). Early life stress enhances the susceptibility to depression and interferes with neuroplasticity in the hippocampus of adolescent mice via regulating miR-34c-5p/SYT1 axis. J Psychiatr Res.

[42] Ge J-F, Qi C-C, Zhou J-N (2013). Imbalance of leptin pathway and hypothalamus synaptic plasticity markers are associated with stress-induced depression in rats. Behav Brain Res.

[43] Müller HK, Wegener G, Popoli M, Elfving B (2011). Differential expression of synaptic proteins after chronic restraint stress in rat prefrontal cortex and hippocampus. Brain Res.

[44] Thome J, Pesold B, Baader M, Hu M, Gewirtz JC, Duman RS (2001). Stress differentially regulates synaptophysin and synaptotagmin expression in hippocampus. Biol Psychiatry.

[45] Kim J, Seol S, Kim T-E, Lee J, Koo JW, Kang HJ (2024). Synaptotagmin-4 induces anhedonic responses to chronic stress via BDNF signaling in the medial prefrontal cortex. Exp Mol Med.

[46] Kayagaki N, Webster JD, Newton K (2024). Control of Cell Death in Health and Disease. Annu Rev Pathol.

[47] Tang D, Kang R, Berghe TV, Vandenabeele P, Kroemer G (2019). The molecular machinery of regulated cell death. Cell Res.

[48] Xu T, Ding W, Ao X, Chu X, Wan Q, Wang Y (2019). ARC regulates programmed necrosis and myocardial ischemia/reperfusion injury through the inhibition of mPTP opening. Redox Biol.

[49] Sun T, Ding W, Xu T, Ao X, Yu T, Li M (2019). Parkin Regulates Programmed Necrosis and Myocardial Ischemia/Reperfusion Injury by Targeting Cyclophilin-D. Antioxid Redox Signal.

[50] Mendoza A, Patel P, Robichaux D, Ramirez D, Karch J (2024). Inhibition of the mPTP and Lipid Peroxidation Is Additively Protective Against I/R Injury. Circ Res.

[51] Duan C, Kuang L, Hong C, Xiang X, Liu J, Li Q (2021). Mitochondrial Drp1 recognizes and induces excessive mPTP opening after hypoxia through BAX-PiC and LRRK2-HK2. Cell Death Dis.

[52] Rohde K, Kleinesudeik L, Roesler S, Löwe O, Heidler J, Schröder K (2017). A Bak-dependent mitochondrial amplification step contributes to Smac mimetic/glucocorticoid-induced necroptosis. Cell Death Differ.

[53] Whelan RS, Konstantinidis K, Wei AC, Chen Y, Reyna DE, Jha S (2012). Bax regulates primary necrosis through mitochondrial dynamics. Proc Natl Acad Sci U S A.

[54] Wu Y, Leng Y, Meng Q, Xue R, Zhao B, Zhan L (2017). Suppression of Excessive Histone Deacetylases Activity in Diabetic Hearts Attenuates Myocardial Ischemia/Reperfusion Injury via Mitochondria Apoptosis Pathway. J Diabetes Res.

[55] Shinde VM, Sizova OS, Lin JH, LaVail MM, Gorbatyuk MS (2012). ER stress in retinal degeneration in S334ter Rho rats. PLoS One.

[56] Wang L, Zhang L, Gong XD, Fu JL, Gan YW, Hou M (2021). PP-1β and PP-2Aα modulate cAMP response element-binding protein (CREB) functions in aging control and stress response through de-regulation of αB-crystallin gene and p300-p53 signaling axis. Aging Cell.

[57] Qin J, Chen HG, Yan Q, Deng M, Liu J, Doerge S (2008). Protein phosphatase-2A is a target of epigallocatechin-3-gallate and modulates p53-Bak apoptotic pathway. Cancer Res.

[58] Du Y, Wang G, Liu B, Guo M, Yan X, Dou M (2024). Naringin alleviates fluoride-induced neurological impairment: A focus on the regulation of energy metabolism mediated by mitochondrial permeability transition pore. Sci Total Environ.

[59] Yu FF, Yu SY, Sun L, Zuo J, Luo KT, Wang M (2024). T-2 toxin induces mitochondrial dysfunction in chondrocytes via the p53-cyclophilin D pathway. J Hazard Mater.

[60] Girod JP, Brotman DJ (2004). Does altered glucocorticoid homeostasis increase cardiovascular risk?. Cardiovasc Res.

[61] Park SR, Kim SK, Kim SR, Kim D, Kim KW, Hong IS (2021). Noncanonical functions of glucocorticoids: A novel role for glucocorticoids in performing multiple beneficial functions in endometrial stem cells. Cell Death Dis.

[62] Nagasawa K, Matsuura N, Takeshita Y, Ito S, Sano Y, Yamada Y (2016). Attenuation of cold stress-induced exacerbation of cardiac and adipose tissue pathology and metabolic disorders in a rat model of metabolic syndrome by the glucocorticoid receptor antagonist RU486. Nutr Diabetes.

[63] Jung S, Choe S, Woo H, Jeong H, An HK, Moon H (2020). Autophagic death of neural stem cells mediates chronic stress-induced decline of adult hippocampal neurogenesis and cognitive deficits. Autophagy.

[64] Wagner M, Moritz A, Volk T (2011). Interaction of gonadal steroids and the glucocorticoid corticosterone in the regulation of the L-type Ca(2+) current in rat left ventricular cardiomyocytes. Acta Physiol (Oxf).

[65] Chetty S, Friedman AR, Taravosh-Lahn K, Kirby ED, Mirescu C, Guo F (2014). Stress and glucocorticoids promote oligodendrogenesis in the adult hippocampus. Mol Psychiatry.

[66] Choi GE, Park JY, Park MR, Yoon JH, Han HJ (2023). Glucocorticoid enhances presenilin1-dependent Aβ production at ER's mitochondrial-associated membrane by downregulating Rer1 in neuronal cells. Redox Biol.

[67] Zhang Y, Jiang ZY, Wang M, Zhang XT, Ge P, Wang W (2024). Helicid Alleviates Neuronal Apoptosis of Rats with Depression-Like Behaviors by Downregulating lncRNA-NONRATT030918.2. Mol Neurobiol.

[68] Zou Z, Xiao N, Chen Z, Lin X, Li Y, Li P (2024). Yeast Extract Peptides Alleviate Depression in Chronic Restraint Stress Rats by Alleviating Hippocampal Neuronal Apoptosis and Dysbiosis of the Gut Microbiota. Mol Nutr Food Res.

[69] Jin W, Xu X, Chen X, Qi W, Lu J, Yan X (2019). Protective effect of pig brain polypeptides against corticosterone-induced oxidative stress, inflammatory response, and apoptosis in PC12 cells. Biomed Pharmacother.

[70] Hyun SA, Lee YJ, Jang S, Ko MY, Lee CY, Cho YW (2022). Adipose stem cell-derived extracellular vesicles ameliorates corticosterone-induced apoptosis in the cortical neurons via inhibition of ER stress. Stem Cell Res Ther.

[71] Xu B, Lang LM, Li SZ, Guo JR, Wang JF, Wang D (2019). Cortisol Excess-Mediated Mitochondrial Damage Induced Hippocampal Neuronal Apoptosis in Mice Following Cold Exposure. Cells.

[72] Chai Y, Cai Y, Fu Y, Wang Y, Zhang Y, Zhang X (2022). Salidroside Ameliorates Depression by Suppressing NLRP3-Mediated Pyroptosis via P2X7/NF-κB/NLRP3 Signaling Pathway. Front Pharmacol.

[73] Zhu Q, Han Y, He Y, Meng P, Fu Y, Yang H (2024). Quercetin inhibits neuronal Ferroptosis and promotes immune response by targeting lipid metabolism-related gene PTGS2 to alleviate breast cancer-related depression. Phytomedicine.

[74] Li E, Yin H, Su M, Li Q, Zhao Y, Zhang L (2023). Inhibition of ferroptosis alleviates chronic unpredictable mild stress-induced depression in mice via tsRNA-3029b. Brain Res Bull.

[75] Shen J, Xie P, Wang J, Yang F, Li S, Jiang H (2024). Nlrp6 protects from corticosterone-induced NSPC ferroptosis by modulating RIG-1/MAVS-mediated mitophagy. Redox Biol.

[76] Zhao M, Ren Z, Zhao A, Tang Y, Kuang J, Li M (2024). Gut bacteria-driven homovanillic acid alleviates depression by modulating synaptic integrity. Cell Metab.

[77] Liang JY, Gao S, Jiang JM, Zhang P, Zou W, Tang XQ (2024). Itaconate inhibits corticosterone-induced necroptosis and neuroinflammation via up-regulating menin in HT22 cells. J Physiol Biochem.

[78] Estades Ayuso V, Pickles S, Todd T, Yue M, Jansen-West K, Song Y (2023). TDP-43-regulated cryptic RNAs accumulate in Alzheimer's disease brains. Mol Neurodegener.

[79] Xia X, Li Y (2025). A high-performance GRAB sensor reveals differences in the dynamics and molecular regulation between neuropeptide and neurotransmitter release. Nat Commun.

[80] Wang P, Tong K, Li Y, Li X, Zhang Y, Gu J (2024). The role and mechanism of HIF-1α-mediated glypican-3 secretion in hypoxia-induced tumor progression in hepatocellular carcinoma. Cell Signal.

[81] Liu X, Li C, Yang Y, Liu X, Li R, Zhang M (2019). Synaptotagmin 7 in twist-related protein 1-mediated epithelial - Mesenchymal transition of non-small cell lung cancer. EBioMedicine.

[82] Li Y, Shao F, Huang Y, Yin Q, Liu J, Zhao Y (2024). SYT7 as a Potential Prognostic Marker Promotes the Metastasis of Epithelial Ovarian Cancer Cells by Activating the STAT3 Pathway. Mol Carcinog.

[83] Huang J, Xu W, Huang Q, Chen E, Chen J (2024). SYT7 (synaptotagmin 7) promotes cervical squamous cell carcinoma. Heliyon.

[84] Zhang W, Zhang J, Wang Z, Li T, Liu C, Kang X (2024). Extracellular RIPK3 Acts as a Damage-Associated Molecular Pattern to Exaggerate Cardiac Ischemia/Reperfusion Injury. Circulation.

[85] Wang H, Guo M, Wei H, Chen Y (2023). Targeting p53 pathways: mechanisms, structures, and advances in therapy. Signal Transduct Target Ther.

[86] Wang K, Liu F, Liu CY, An T, Zhang J, Zhou LY (2016). The long noncoding RNA NRF regulates programmed necrosis and myocardial injury during ischemia and reperfusion by targeting miR-873. Cell Death Differ.

[87] Zhou J, Qin X, Li L, Tian D, Zou Z, Gu Z (2023). Heat stress-induced intestinal epithelial cells necroptosis via TLR3-TRIF-RIP3 pathway was dependent on p53. Int Immunopharmacol.

[88] Dashzeveg N, Yoshida K (2015). Cell death decision by p53 via control of the mitochondrial membrane. Cancer Lett.

[89] Patel P, Mendoza A, Robichaux DJ, Wang MC, Wehrens XHT, Karch J (2021). Inhibition of the Anti-Apoptotic Bcl-2 Family by BH3 Mimetics Sensitize the Mitochondrial Permeability Transition Pore Through Bax and Bak. Front Cell Dev Biol.

[90] Flores-Romero H, Dadsena S, García-Sáez AJ (2023). Mitochondrial pores at the crossroad between cell death and inflammatory signaling. Mol Cell.

[91] Tu HC, Ren D, Wang GX, Chen DY, Westergard TD, Kim H (2009). The p53-cathepsin axis cooperates with ROS to activate programmed necrotic death upon DNA damage. Proc Natl Acad Sci U S A.

