# Supplementary material for: Synaptotagmin-7 drives stress-induced cardiomyocyte necroptosis via the p53-Bak-mPTP axis
Source: Theranostics. 2026 Jan 1;16(5):2517–37. doi: 10.7150/thno.119528 (PMC12712958; doi:10.7150/thno.119528)
Supplement: Supplementary file 1 — Supplementary figures. [file thnov16p2517s1.pdf]

Supplementary Figures and figure legends

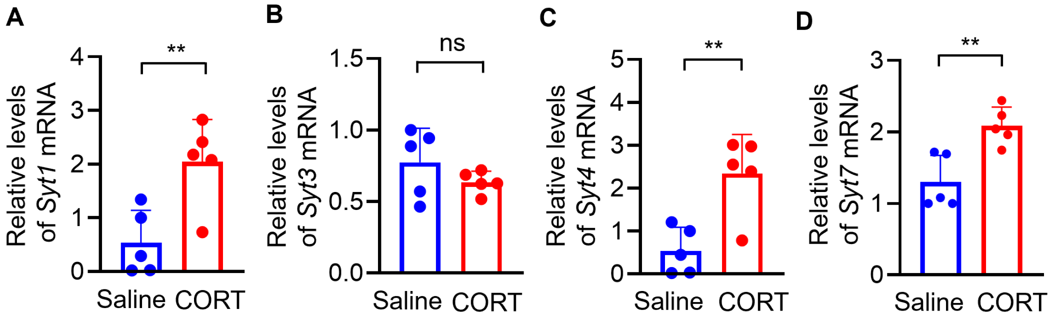

Supplementary Figure S1. The expression levels of Synaptotagmin family members (*Syt1*, *Syt3*, *Syt4*, and *Syt7*) in CORT-treated mouse hearts. Wild-type (WT) mice received daily intraperitoneal injections of corticosterone (CORT, 10 mg/kg) or an equivalent volume of saline for 7 days. Cardiac mRNA expression levels were assessed by RT-qPCR. **A.** Syt1 mRNA levels. ** $p < 0.01$. n = 5. **B.** Syt3 mRNA levels. ns, not significant. n = 5. **C.** Syt4 mRNA levels. ** $p < 0.01$. n = 5. **D.** Syt7 mRNA levels. ** $p < 0.01$. n = 5. Data are expressed as mean ± standard deviation (SD).

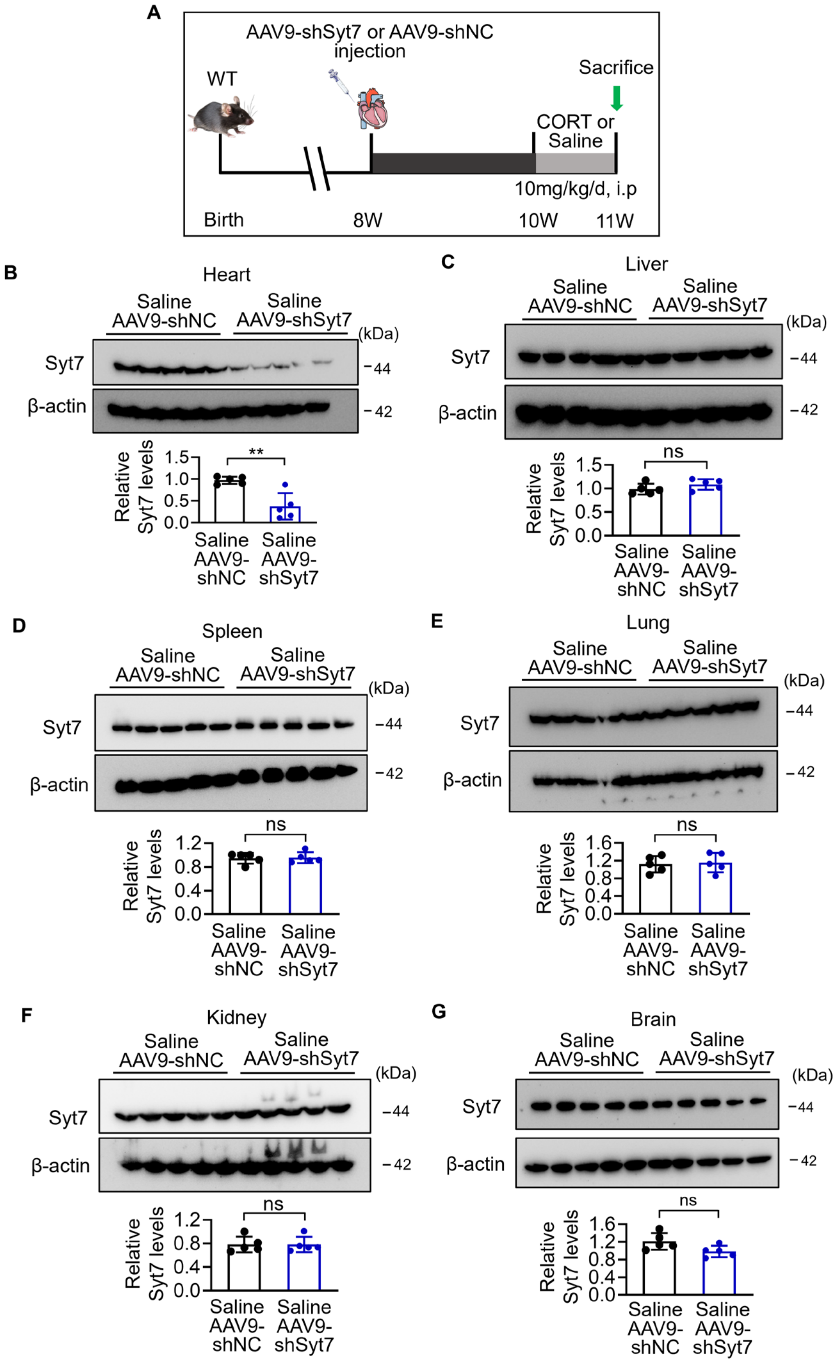

Supplementary Figure S2. Evaluation of Syt7 expression in cardiac and non-cardiac tissues following AAV9-sh*Syt7* administration. A. Schematic diagram of the experimental design. Wild-type (WT) mice received *in situ* myocardial injections of AAV9-sh*Syt7* or AAV9-shNC, and two weeks later were administered daily intraperitoneal injections of corticosterone (CORT, 10 mg/kg) or saline for 7 consecutive days. **B.** Syt7 protein expression levels in heart tissue. ** $p < 0.01$. n = 5. **C-G.** Syt7 protein expression levels in liver tissue (C), spleen tissue (D), lung tissue (E), kidney tissue (F) and brain tissue (G). ns, not significant. n = 5. Data are expressed as mean ± standard deviation (SD).

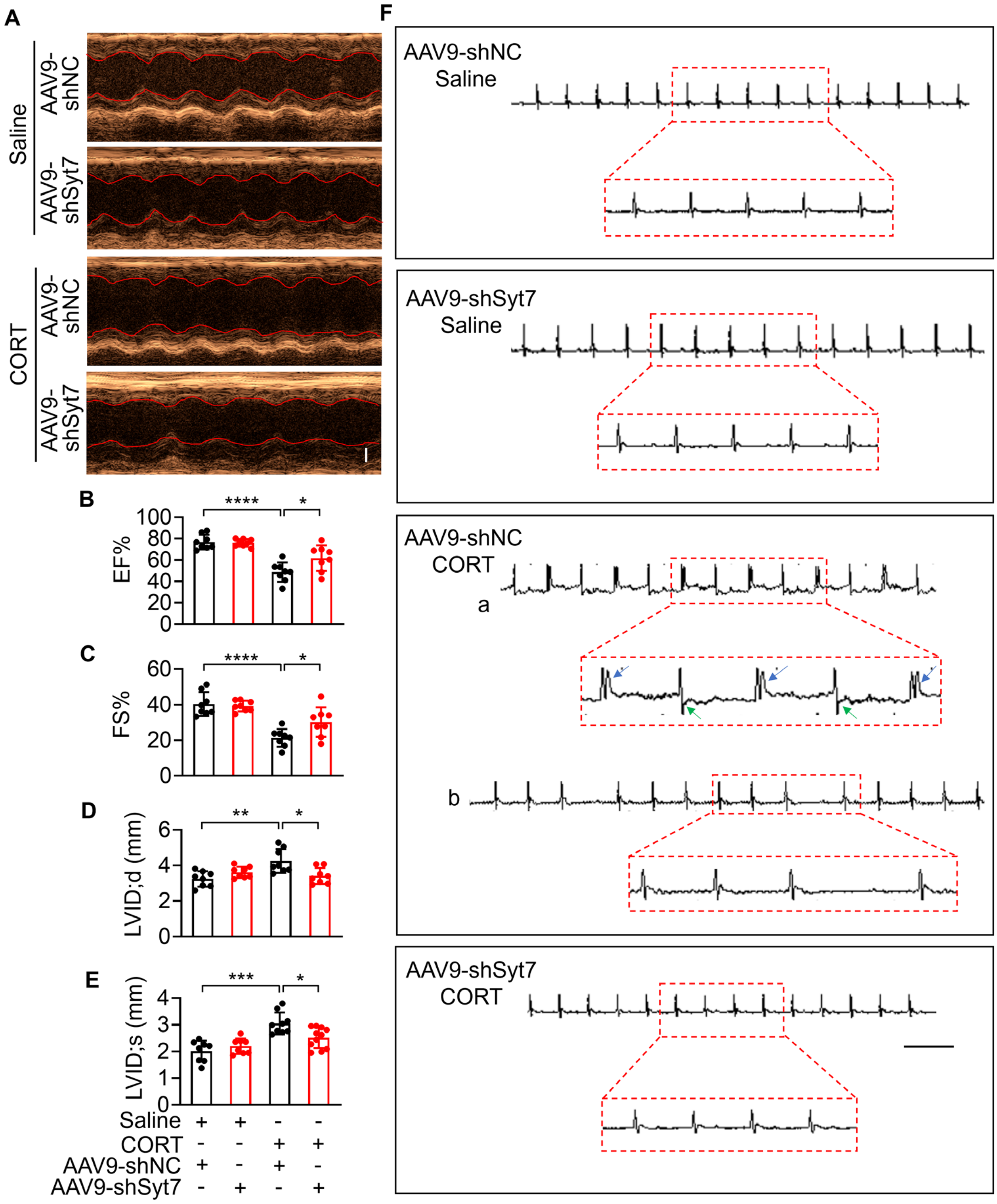

Supplementary Figure S3. Cardiomyocyte-specific knockdown of *Syt7* attenuates CORT-induced cardiac structural and electrophysiological remodeling. A-E. Representative echocardiographic images (A) and quantitative analysis of cardiac function parameters including ejection fraction (EF) (B), fractional shortening (FS) (C), left ventricular internal diameter in diastole (LVID;d) (D), and in systole (LVID;s) (E). Bar = 1 mm. * $p < 0.05$. ** $p < 0.01$. *** $p < 0.001$. **** $p < 0.0001$. n = 8. **F.** Representative electrocardiograms. Bar = 100 ms. Data are expressed as mean ± standard deviation (SD).

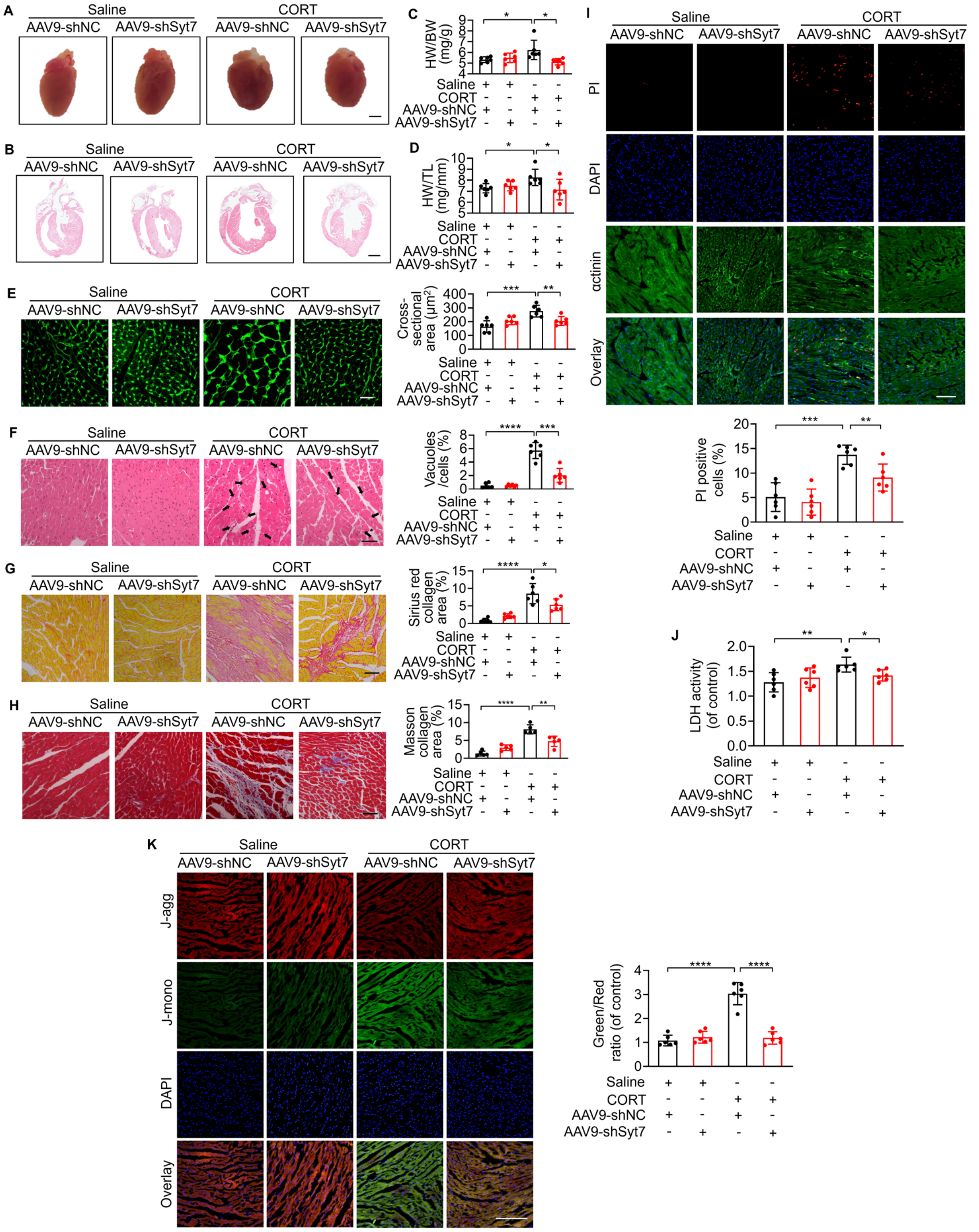

Supplementary Figure S4. Cardiomyocyte-specific *Syt7* knockdown alleviates CORT-induced myocardial injury and necroptosis. A-B. Representative gross heart images and longitudinal sections stained with hematoxylin and eosin (H&E). Bar = 2 mm. **C-D.** Quantification of heart weight to body weight ratio (HW/BW, mg/g) and heart weight to tibia length ratio (HW/TL, mg/mm). * $p < 0.05$. n = 6. **E.** Cross-sectional area of cardiomyocytes evaluated by wheat germ agglutinin (WGA) staining. Bar = 20 μm. ** $p < 0.01$. *** $p < 0.001$. n = 6. **F.** Vacuolar degeneration in cardiomyocytes detected by H&E staining. Arrows indicate vacuolar degeneration. Bar = 50 μm. *** $p < 0.001$. **** $p < 0.0001$. n = 6. **G.** Myocardial fibrosis evaluated by Sirius Red staining. Bar = 50 μm. * $p < 0.05$. **** $p < 0.0001$. n = 6. **H.** Myocardial fibrosis assessed by Masson's trichrome staining. Bar = 50 μm. ** $p < 0.01$. **** $p < 0.0001$. n = 5. **I.** Cardiomyocyte necrosis detected by PI staining. Red: PI; blue: DAPI; green: α-actinin. Bar = 50 μm. ** $p < 0.01$. *** $p < 0.001$. n = 6. **J.** LDH activity detection. * $p < 0.05$. ** $p < 0.01$. n = 6. **K.** Mitochondrial membrane potential

(ΔΨm) determined by JC-1 staining, calculated as the ratio of J-monomers to J-aggregates. Bar = 100 μm. **** $p < 0.0001$. n = 6. Data are expressed as mean ± standard deviation (SD).

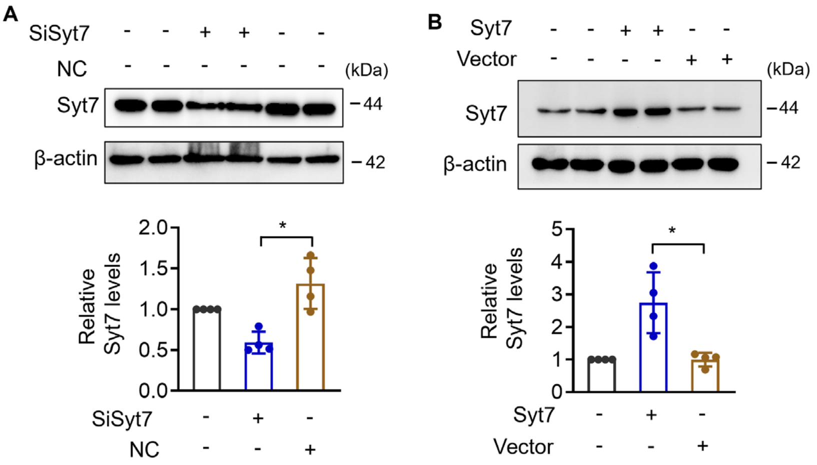

Supplementary Figure S5. Verification of Syt7 expression following Si*Syt7* or *Syt7* overexpression. A. Western blot analysis of Syt7 protein levels in H9c2 cells after 48 h treatment with Si*Syt7* or negative control (NC). * $p < 0.05$. n = 4. **B.** Western blot analysis of Syt7 protein levels in H9c2 cells following overexpression of *Syt7* for 48 h. * $p < 0.05$. n = 4. Data are expressed as mean ± standard deviation (SD).

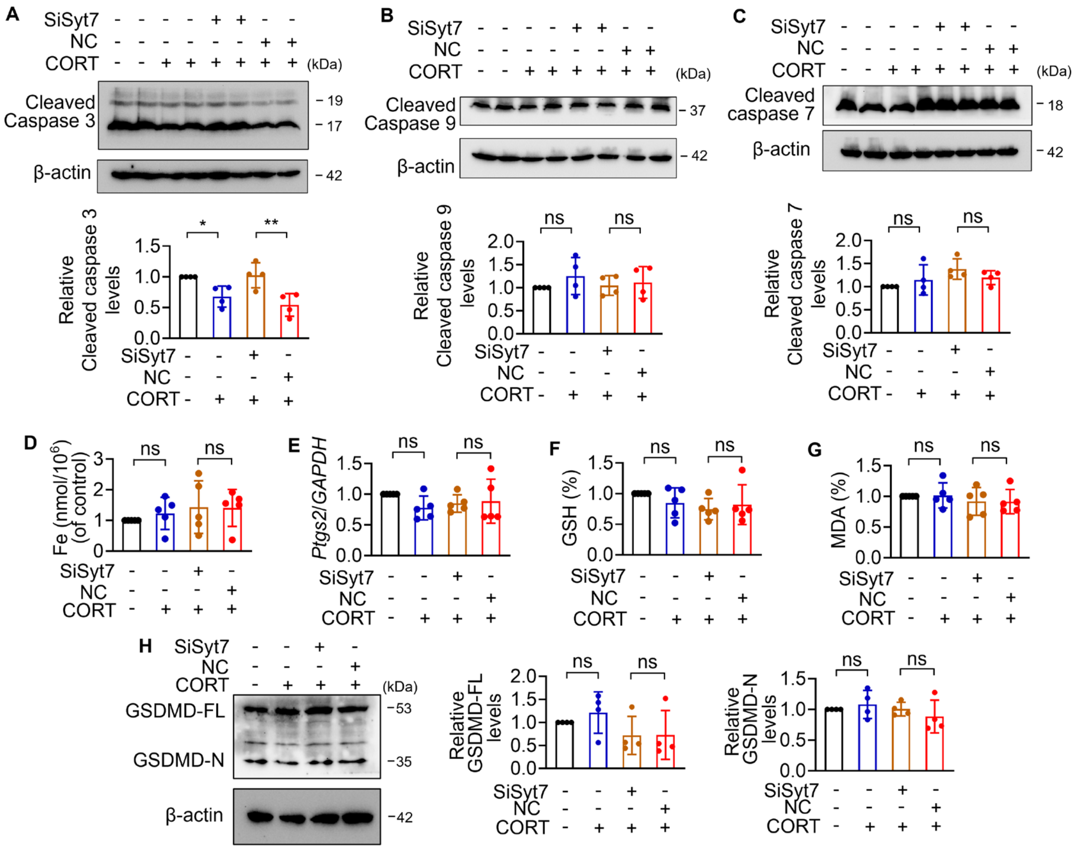

Supplementary Figure S6. Evaluation of classical apoptosis, ferroptosis, and pyroptosis pathways in CORT-induced myocardial injury and the role of *Syt7*. H9c2 cells were infected with *Syt7* siRNA adenovirus or negative control (NC) and treated with 1 μM CORT for 48 h. **A.** Cleaved caspase-3 protein levels. * $p < 0.05$. ** $p < 0.01$. n = 4. **B.** Cleaved caspase-9 protein levels. ns, not significant. n = 4. **C.** Cleaved caspase-7 protein levels. ns, not significant. n = 4. **D.** Relative intracellular iron levels. ns, not significant. n = 5. **E.** The relative mRNA levels of ferroptosis marker *Ptgs2*. ns, not significant. n = 5. **F.** Relative glutathione (GSH) levels. ns, not significant. n = 5. **G.** Relative Malondialdehyde (MDA) levels. ns, not significant. n = 5. **H.** Protein levels of full-length GSDMD (GSDMD-FL) and cleaved GSDMD (GSDMD-N). ns, not significant. n = 4. Data are expressed as mean ± standard deviation (SD).

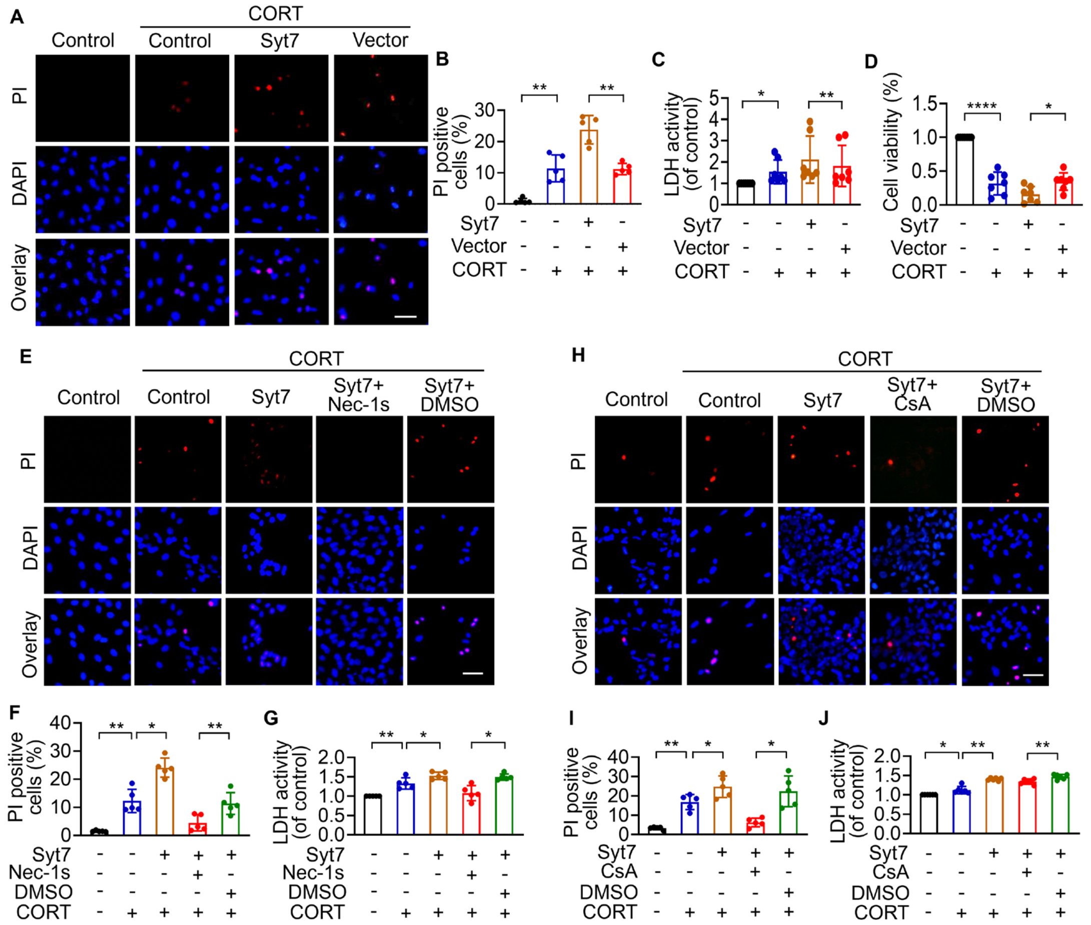

Supplementary Figure S7. Cardiomyocyte vulnerability to CORT-induced necrosis following *Syt7* overexpression. A-D. H9c2 cells were transfected with *Syt7* overexpression plasmids, and then exposed to 1 μM CORT for 48 h. **A-B.** PI staining. Bar = 50 μm. ** $p < 0.01$. n = 5. **C.** LDH activity detection. * $p < 0.05$. ** $p < 0.01$. n = 7. **D.** Cell viability. * $p < 0.05$. **** $p < 0.0001$. n = 7. **E-G.** Quantification of PI-positive cells (E-F) and LDH activity (G) with or without Nec-1s (necrosis inhibitor) treatment. Bar = 50 μm. * $p < 0.05$. ** $p < 0.01$. n = 5. **H-J.** Quantification of PI-positive cells (H-I, n = 5) and LDH activity (J, n = 6) with or without CsA (mPTP inhibitor) treatment. Bar = 50 μm. * $p < 0.05$. ** $p < 0.01$. Data are expressed as mean ± standard deviation (SD).

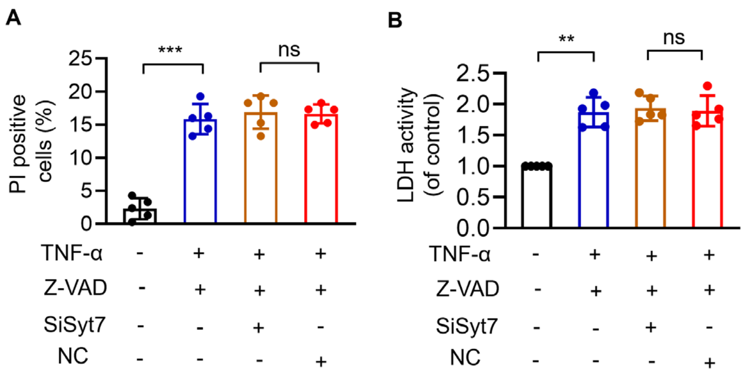

Supplementary Figure S8. Syt7 does not mediate classical death receptor-dependent necroptosis. H9c2 cells were treated with tumor necrosis factor-α (TNF-α) and z-VAD for 48 h in the presence or absence of *Syt7* siRNAs. **A.** PI staining. *** $p < 0.001$. ns, not significant. n = 5. **B.** LDH activity assay. ** $p < 0.01$. ns, not significant. n = 5. Data are expressed as mean ± standard deviation (SD).

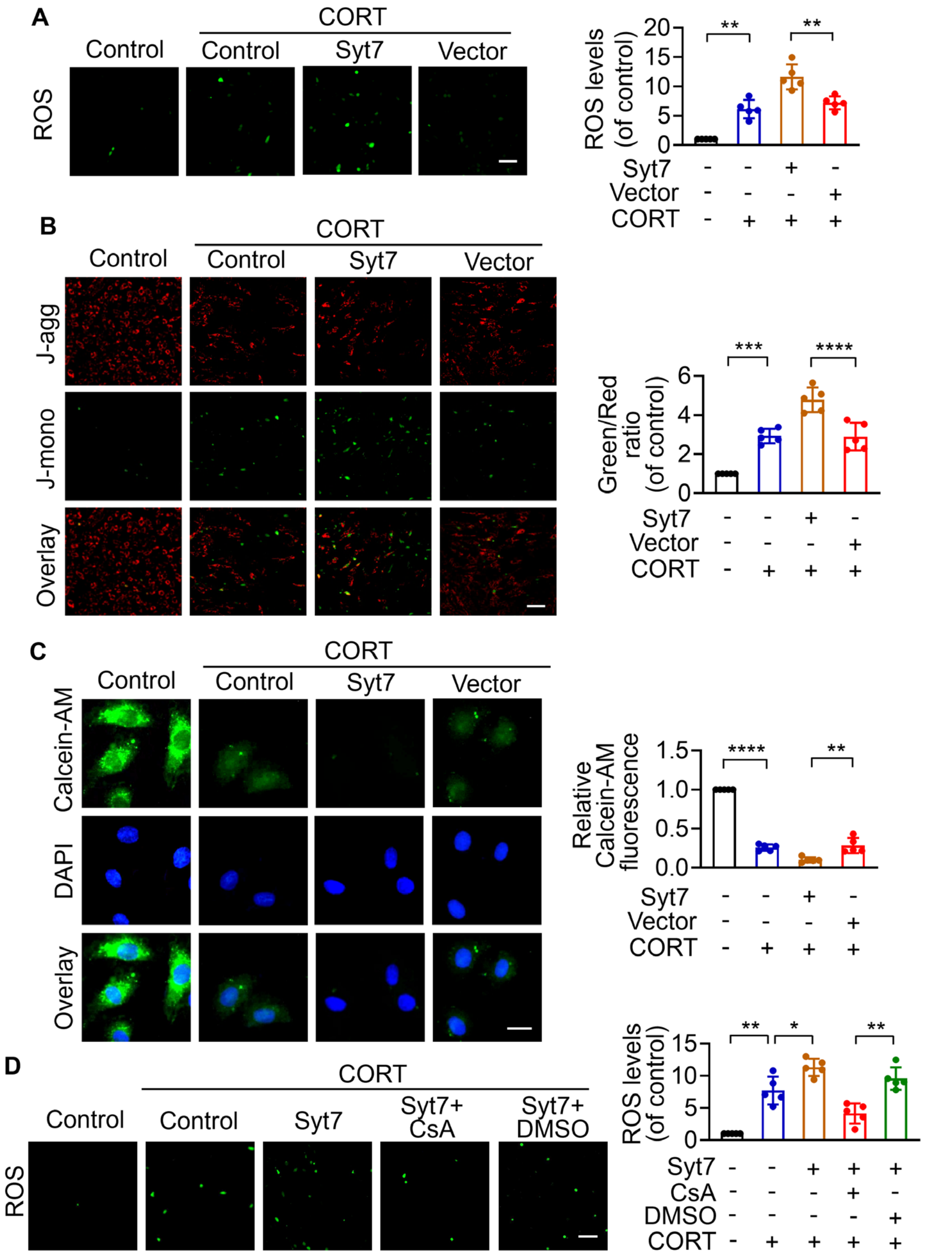

Supplementary Figure S9. Syt7 enhances CORT-induced mPTP opening in cardiomyocytes. A-C. H9c2 cells were transfected with *Syt7* overexpression plasmids, followed by treatment with 1 μM CORT for 48 h. **A.** ROS levels detected using DCFH-DA probe. Bar = 100 μm. ** $p < 0.01$. n = 5. **B.** Mitochondrial membrane potential (ΔΨm) assessed by 5,5',6,6'-tetrachloro-1,1',3,3'-tetraethyl-1H-imidazole-2-carboxylic acid (JC-1) in cardiomyocytes. Ratio of J-monomers to J-aggregates calculated. Bar = 50 μm. *** $p < 0.001$. **** $p < 0.0001$. n = 5. **C.** mPTP opening assessed by Calcein-AM staining. Bar = 20 μm. ** $p < 0.01$. **** $p < 0.0001$. n = 5. **D.** H9c2 cells were transfected with *Syt7* overexpression plasmids, and then treated with 1 μM CORT in the presence or absence of CsA (mPTP inhibitor) for 48 h. ROS levels were detected using DCFH-DA probe. Bar = 100 μm. * $p < 0.05$. ** $p < 0.01$. n = 5. Data are expressed as mean ± standard deviation (SD).

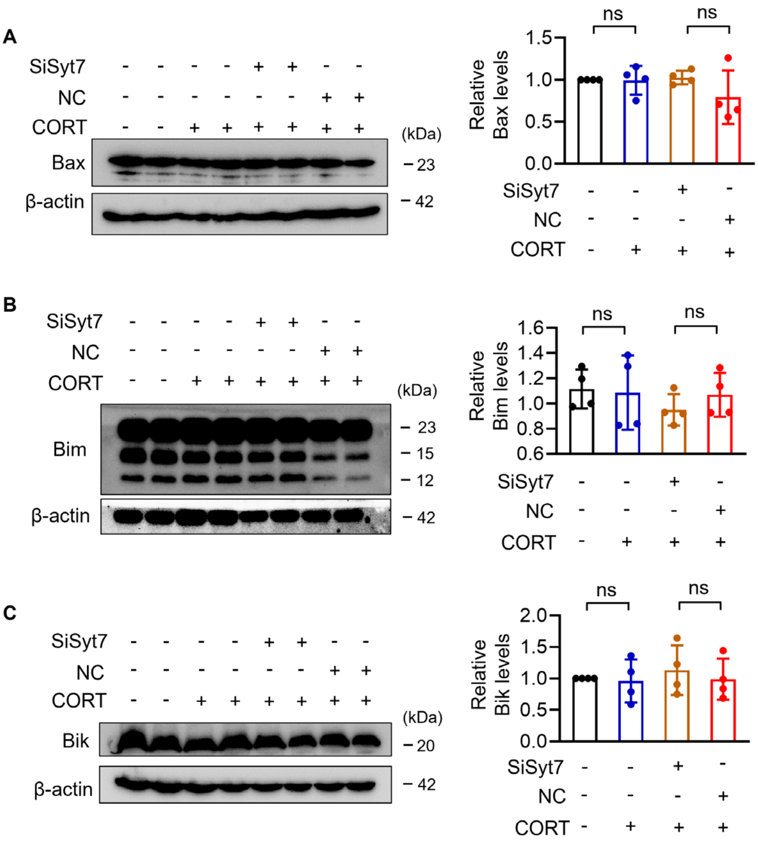

Supplementary Figure S10. Expression levels of key regulators involved in mPTP opening. A. Bcl-2 associated X protein (Bax) protein levels. ns, not significant. n = 4. **B.** Bcl-2 interacting mediator of cell death (Bim) protein levels. ns, not significant. n = 4. **C.** Bcl-2 interacting killer (Bik) protein levels. ns, not significant. n = 4. Data are expressed as mean ± standard deviation (SD).

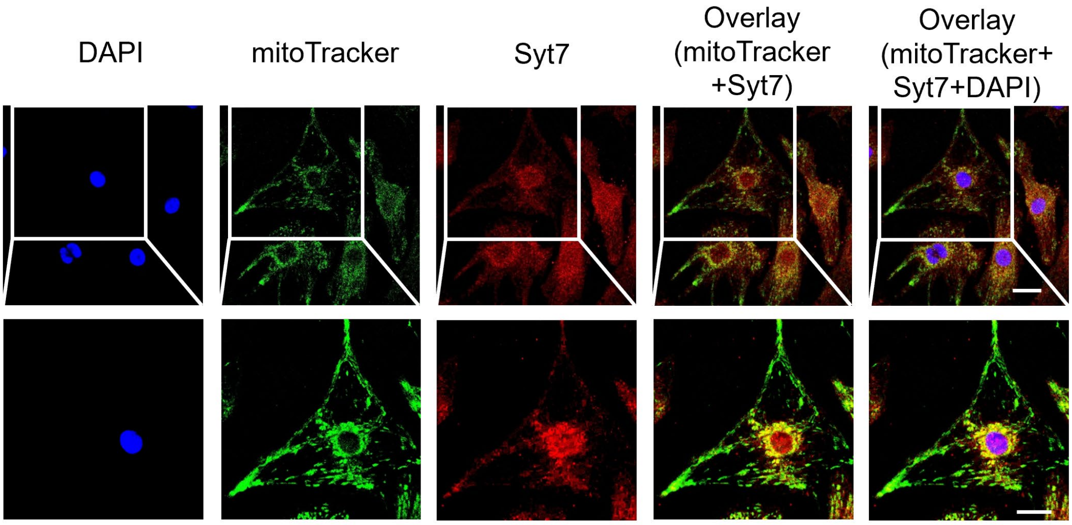

Supplementary Figure S11. Sub-cellular localization of Syt7 in cardiomyocytes. Neonatal rat primary cardiomyocytes were isolated and labelled with DAPI and Mito-Tracker to visualize the intracellular distribution of Syt7. Syt7 (red); Mitochondria (green, MitoTracker); Nuclei (blue, DAPI). Top panel: Bar = 25 µm. Bottom panel: Bar = 20 µm.

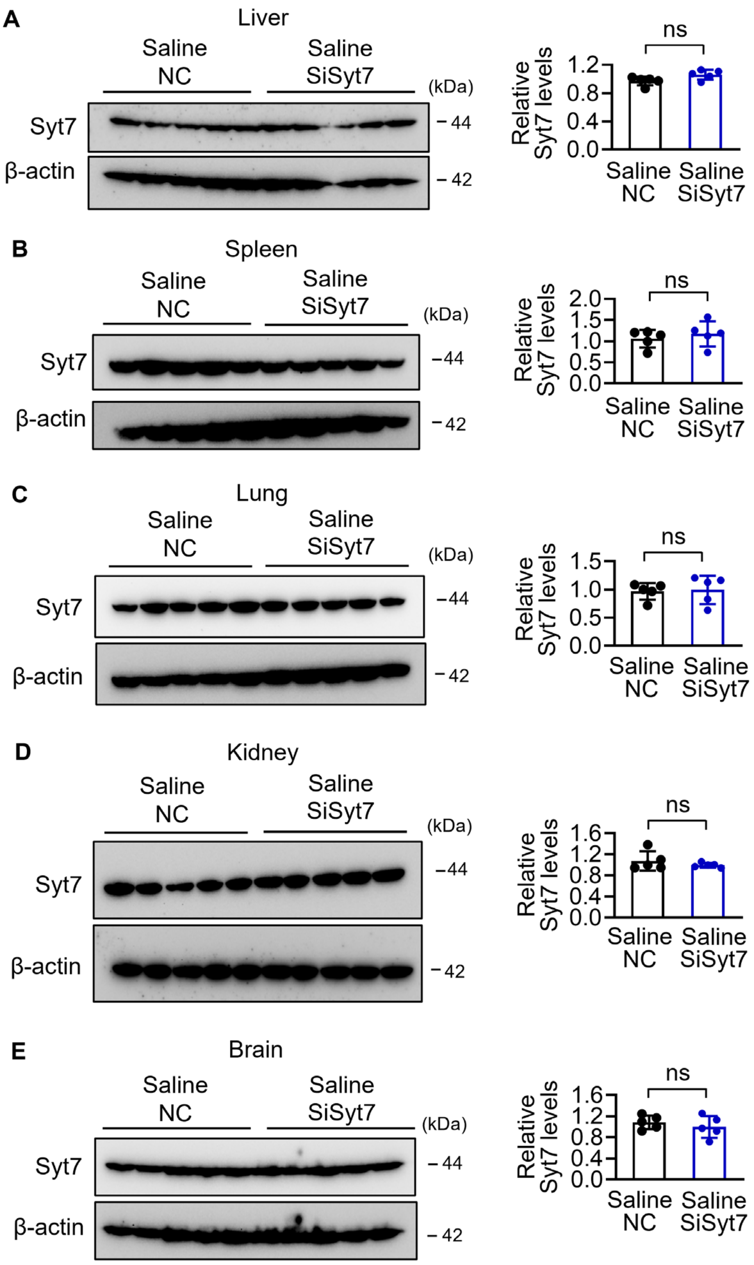

Supplementary Figure S12. Syt7 expression in non-cardiac tissues following *Syt7* siRNA adenovirus administration. The Syt7 protein levels in liver tissue (A), spleen tissue (B), lung tissue (C), kidney tissue (D) and brain tissue (E). ns, not significant. n = 5. Data are expressed as mean ± standard deviation (SD).
